# Cytosine base editors (CBEs) for inducing targeted DNA base editing in *Nicotiana benthamiana*

**DOI:** 10.1186/s12870-023-04322-8

**Published:** 2023-06-07

**Authors:** Juan Luo, Muhammad Abid, Jing Tu, Xinxia Cai, Yi Zhang, Puxin Gao, Hongwen Huang

**Affiliations:** 1 Lushan Botanical Garden, Chinese Academy of Sciences, Jiujiang, 332900 China; 2 College of Life Science, Nanchang University, Nanchang, 330031 China

**Keywords:** CRISPR, Cytosine base editor, A3A, A3A(Y130F), rAPOBEC1(R33A), Polyploid plants

## Abstract

**Background:**

The base editors can introduce point mutations accurately without causing double-stranded DNA breaks or requiring donor DNA templates. Previously, cytosine base editors (CBEs) containing different deaminases are reported for precise and accurate base editing in plants. However, the knowledge of CBEs in polyploid plants is inadequate and needs further exploration.

**Results:**

In the present study, we constructed three polycistronic tRNA-gRNA expression cassettes CBEs containing A3A, A3A (Y130F), and rAPOBEC1(R33A) to compare their base editing efficiency in allotetraploid *N. benthamiana* (*n* = 4x). We used 14 target sites to compare their editing efficiency using transient transformation in tobacco plants. The sanger sequencing and deep sequencing results showed that A3A-CBE was the most efficient base editor. In addition, the results showed that A3A-CBE provided most comprehensive editing window (C_1_ ~ C_17_ could be edited) and had a better editing efficiency under the base background of TC. The target sites (T2 and T6) analysis in transformed *N. benthamiana* showed that only A3A-CBE can have C-to-T editing events and the editing efficiency of T2 was higher than T6. Additionally, no off-target events were found in transformed *N. benthamiana*.

**Conclusions:**

All in all, we conclude that A3A-CBE is the most suitable vector for specific C to T conversion in *N. benthamiana*. Current findings will provide valuable insights into selecting an appropriate base editor for breeding polyploid plants.

**Supplementary Information:**

The online version contains supplementary material available at 10.1186/s12870-023-04322-8.

## Background

Gene editing tools, especially the clustered regularly interspaced short palindromic repeat (CRISPR) systems, have shown attractive prospects in plant breeding since their advent [1]. Single nucleotide polymorphisms (SNPs) are significantly related to plant agronomic characteristics and are an essential direction of molecular breeding [2–4]. The CRISPR system needs a donor template to induce a homology-directed repair pathway (HDR) for an accurate gene editing [5]. However, the efficiency of HDR is very low, which hinders its application in plants [6]. The base editing can convert DNA bases directly at the target site, which provides an exciting tool for SNPs-based plant breeding [7].

Base editors, structurally composed of base modification enzymes and defective catalytic Cas9, can be divided into cytosine base editor (CBE) and adenine base editor (ABE) [8]. The cytosine deaminase in CBE first catalyzes cytosine (C) to uracil (U), then reads as thymine (T) during DNA replication [7]. Similarly, adenine deaminase in ABE first catalyzes adenine (A) to inosine (I), then reads as guanine (G) during DNA replication [9]. Previous studies have found that adding uracil glycosylase inhibitor (UGI) to CBE can reduce the U excision rate and improve C to T editing efficiency [10]. Base editors are safer than the CRISPR system and have broader application prospects in plant breeding because they do not form double-strand breaks (DSBs) [7].

Although the editing efficiency of the third generation CBE (CBE3), which harbors a rat APOBEC1 (rAPOBEC1) enzyme, is higher than that of HDR, it is still necessary to assess and improve their editing efficiency for different plant species and different targets [11]. The previous study had shown that CBE3 was constructed by rAPOBEC1 mutations [rAPOBEC1(R33A)], namely rAPOBEC1(R33A)-CBE3, which had the same editing efficiency in the target DNA, but the editing efficiency in RNA was significantly reduced compared with rAPOBEC1-CBE3 [12]. A comparison of the five deaminases rAPOBEC1, human AID (hAID), *Petromyzon marinus* CDA1 (PmCDA1), human APOBEC3A (A3A), and one A3A mutant [A3A(Y130F)] in tomato showed that the CBE constructed by A3A(Y130F) had the highest editing efficiency [13]. Similar studies had demonstrated that the CBE constructed by A3A(Y130F) had the highest editing efficiency in *Oryza sativa* and *Arabidopsis thaliana* by comparing seven different deaminases [14]. Another study had shown that A3A (can convert all C in the edit window) was more suitable than rAPOBEC1 (can convert a maximum of five C in the edit window) in wheat, rice, and potatoes [15].

Recruiting more UGIs by editors can improve editing efficiency and product purity [14]. However, the comparison of A3A, A3A(Y130F), and rAPOBEC1(R33A) deaminase when recruiting two UGIs has not been studied yet. In the present study, we compared the editing efficiency of A3A, A3A(Y130F), and rAPOBEC1(R33A) when recruiting two UGIs with 14 target sites in model plant *N. benthamiana*. Our findings will provide valuable insights into selecting a suitable editor for increasing the editing efficiency of SNPs based on plant breeding.

## Results

### A3A induced higher C-to-T conversion than A3A(Y130F) and rAPOBEC1(R33A)

**Fig. 1:**
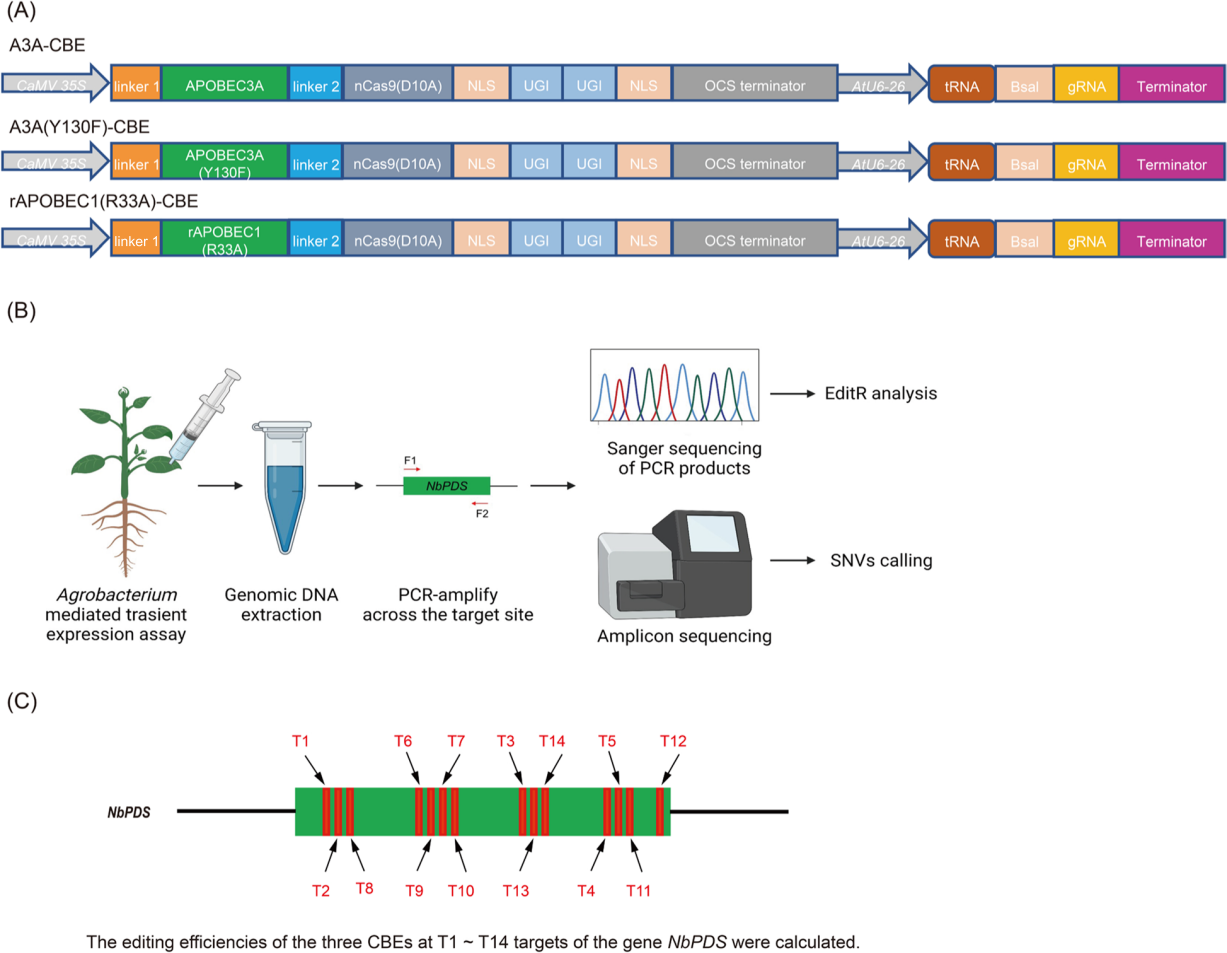
A summary of C-to-T base editing by different CBEs in *N. benthamiana*. **A** The schematic diagrams of A3A-CBE, A3A (Y130F)-CBE, and rAPOBEC1(R33A)-CBE. The detail base sequences of the three CBEs were shown in Figures S8, S9, and S10. **B** Flowchart for *Agrobacterium-*mediated transient overexpression of different target sites in *NbPDS* in tobacco leaves and identification of base editing through sanger sequencing and deep sequencing. F1 indicated forward primer and F2 represented reverse primer for PCR analysis. **C** Schematic diagram indicated the position of 14 sgRNAs on the target *NbPDS* gene. The results showed that the editing efficiencies of A3A-CBE were the highest on T1 ~ T14 in *N. benthamiana*

To compare the editing efficiencies of different cytosine deaminases on plants, we first constructed three different CBE binary vectors (Fig. 1A) containing polycistronic tRNA-gRNA expression cassette (PTG). Then, we designed 14 gRNAs for the target gene *NbPDS* (Fig. 1C) to assess the editing efficiencies of the three CBE binary vectors in tobacco leaves. We introduced the CBE binary vectors into tobacco leaves by *Agrobacterium-mediated* transient overexpression analysis. The *Agrobacterium* host cells containing CBE binary vectors were resuspended in MES buffer to maintain the OD_600_ = 0.5 and were injected into tobacco leaves. The leaves injected with bacterial culture were harvested after 72 h, and genomic DNA was assessed for base conversion after sequencing (Fig. 1B).

**Fig. 2:**
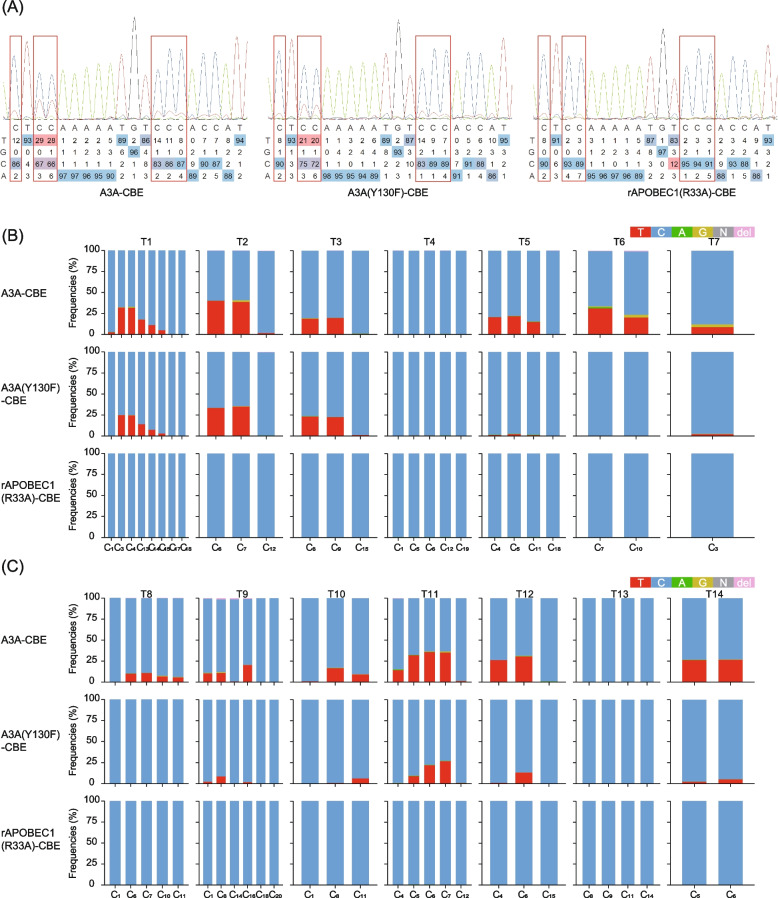
Editing efficiency estimation of binary vectors A3A-CBE, A3A (Y130F)-CBE, and rAPOBEC1(R33A)-CBE in *N. benthamiana*. **A** Sanger sequencing peak map of the T1 target site for CBE binary vectors. The red boxes represent the edited base induced by CBEs. **B** Deep sequencing bar plots of T1 ~ T7 target site for CBE binary vectors. **C** Deep sequencing bar plots of T8 ~ T14 target site for CBE binary vectors. The editing efficiencies were calculated from three independent replicates

The target region including all target sites of the *NbPDS* gene was amplified with specific pair of primers (Table S2). The PCR products were then sent to the company for sanger sequencing, and the EditR software was used to identify gene editing events [16]. The results showed that all CBEs can successfully induce the conversion from C to T. To estimate the editing efficiency of CBE binary vectors accurately, the amplified target region of the *NbPDS* gene was subjected to deep sequencing. The batch search mode in CRISPResso2 software was used to detect a base change in amplicons [17]. The editing efficiency of three CBE binary vectors in 12 target sites was A3A-CBE > A3A(Y130F)-CBE > rAPOBEC1(R33A)-CBE. It was worth noting that all CBE binary vectors had no editing events for both T4 and T13 target sites (Fig. 2). Additionally, the deep sequencing results showed that there were not only conversions from C to T (Figure S1) but also from C to G (Figure S2), C to A (Figure S3), and C to deletion (Figure S4), which was at par with a previously reported study [10]. We obtained very reliable deep sequencing results with few unknown bases in the detection region (Figure S5). The editing efficiency of A3A-CBE for converting C to T (range: 0.01 ~ 40.13%, mean: 12.33%) was 2.27-fold higher than that of A3A(Y130F)-CBE (range: 0.00 ~ 34.98%, mean: 5.43%), while the efficiency of rAPOBEC1(R33A)-CBE was negligible (range: 0.00 ~ 0.29%, mean: 0.03%), indicating that A3A-CBE was the most efficient base editor for *N. benthamiana* plants (Fig. 2 and Figure S1). The editing efficiency of A3A-CBE in converting C to G was 0.36% (range: 0.00 ~ 3.16%), A3A(Y130F)-CBE was 0.05% (range: 0.00 ~ 0.39%), and rAPOBEC1(R33A)-CBE was 0.01% (range: 0.00 ~ 0.05%) (Figure S2). The editing efficiency for conversion of C to A by A3A-CBE was 0.16% (range: 0.01 ~ 1.45%), A3A(Y130F)-CBE was 0.10% (range: 0.01 ~ 0.57%), and rAPOBEC1(R33A)-CBE was 0.09% (range: 0.01 ~ 0.57%) (Figure S3). The editing efficiency for conversion of C to deletion by A3A-CBE was 0.24% (range: 0.00 ~ 1.69%), A3A(Y130F)-CBE was 0.02% (range: 0.00 ~ 0.19%), and rAPOBEC1(R33A)-CBE was 0.00% (range: 0.00 ~ 0.02%) (Figure S4). A3A-CBE also showed the highest non-target base editing and deletions rates, which were quite low. The editing efficiencies of *slEF1ɑ*-A3A-CBE were lower or similar than A3A-CBE among the 14 targets, except T7 target (Figure S6).

### Analysis of editing window and base editing preference

**Fig. 3:**
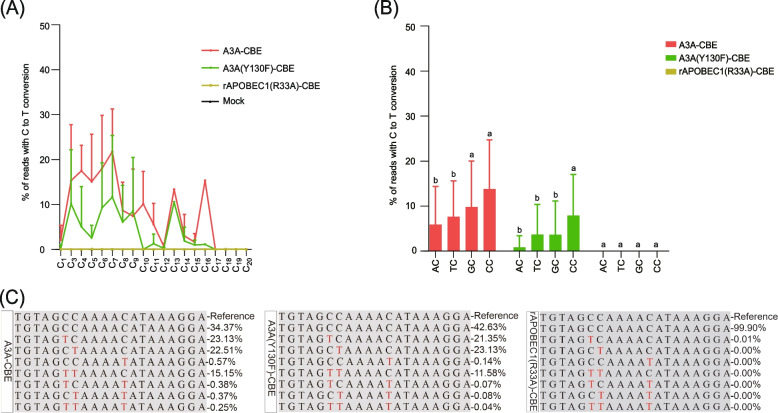
Comparison of editing window, editing backgrounds, and editing products for A3A-CBE, A3A (Y130F)-CBE, and rAPOBEC1(R33A)-CBE. **A** The editing window for CBEs to induce C-to-T conversion. Mock, no treatment. **B** The effect of base background of each NC target site on editing efficiency. **C** The deep sequencing amplicons of the T2 target site induced by A3A-CBE, A3A (Y130F)-CBE, and rAPOBEC1(R33A)-CBE

Further, we compared the editing windows of A3A-CBE, A3A(Y130F)-CBE, and rAPOBEC1(R33A)-CBE. Previously, researchers had reported a massive change in the CBE editing windows. For an instance, it worked from protospacer positions 1 to 20, and more efficient from protospacer positions 3 to 10 in human cells [18]. In rice cells, editing occurred from protospacer position 1 to 18, and the efficiency of the same locus varied significantly with different genes [14]. The editing window of CBE for fourteen target sites of the *NbPDS* gene ranged between protospacer positions 1 ~ 17, and the editing efficiencies of A3A-CBE and A3A(Y130F)-CBE were significantly higher than that of rAPOBEC1(R33A)-CBE at different sites (Fig. 3A). In most target sites, the editing efficiency of A3A-CBE was better than that of A3A(Y130F)-CBE (Fig. 3A), it proved again by the details of T2 target site induced by three CBE binary vectors (Fig. 3C). Previous studies found that the editing efficiency was related to the base background and followed the order TC > CC ≥ AC > GC (the second nucleotide C is the target nucleotide) [11, 19]. However, in the current study, we had observed the following order for editing efficiency: CC > GC > TC > AC (Fig. 3B and Figure S7). Overall, the editing efficiency of CBE binary vector in *N. benthamiana* was related not only to the positions but also to the background of the base.

### A3A-CBE binary vector induced C to T conversion in transgenic tobacco plants

**Fig. 4:**
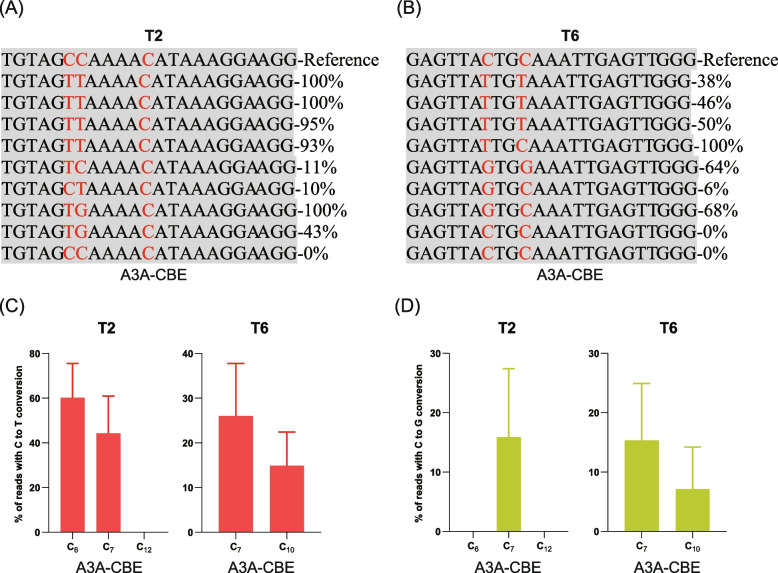
The editing efficiency of A3A-CBE in transformed tobacco. **A** the editing product of T2 target site, **B** the editing product of T6 target site, **C** the editing efficiency for conversion from C-to-T of targets, and **D** the editing efficiency for conversion from C-to-G of targets. The percentages in (**A**) and (**B**) represented the editing efficiency of every sample

We performed stable transformation for T2 and T6 target sites in tobacco plants to compare C to T conversion efficiency of A3A-CBE, A3A(Y130F)-CBE, and rAPOBEC1(R33A)-CBE (Fig. 1C). The genomic DNA extracted from transgenic tobacco plants was used as a PCR template to amplify the *NbPDS* gene using a specific pair of primers (Table S2). The results showed that only A3A-CBE binary vector successfully induced C to T conversion in transgenic plants (Table 1). For the target site T2, 8/9 (88.89%), 0/8 (0.00%), and 0/12 (0.00%) transgenic plants showed C to T conversions for A3A-CBE, A3A(Y130F)-CBE, and rAPOBEC1(R33A)-CBE, respectively (Table 1). For the target site T6, 4/9 (44.44%), 0/10 (0.00%), and 0/11 (0.00%) transgenic plants exhibited C to T conversions for A3A-CBE, A3A(Y130F)-CBE and rAPOBEC1(R33A)-CBE, respectively (Table 1). The A3A(Y130F)-CBE and rAPOBEC1(R33A)-CBE binary vector failed to induce C to T conversion in both target sites. The results of T2 target site for A3A-CBE binary vector showed that three and five transgenic plants were homozygous and heterozygous, respectively. Similarly, the results of target site T6 for A3A-CBE binary vector exhibited that one and three transgenic plants were homozygous and heterozygous, respectively (Table 1). Interestingly, A3A-CBE binary vector base editor induced C to G conversion instead of C to T conversion in some transgenic plants which was an unusual phenomenon, and it would be interesting to further investigate for important agronomic traits in plants (Fig. 4).

**Table 1:** The editing efficiencies of A3A-CBE, A3A (Y130F)-CBE, and rAPOBEC1(R33A)-CBE for T2 and T6 target sites in transformed tobacco

Base editor	Targets	The number of trans genic plants	The number of plants with C-to-T conversion	C-to-T editing efficiency	Heterozygous/Homozygous
A3A-CBE	T2	9	8	88.89%	5/3
	T6	9	4	44.44%	3/1
A3A(Y130F)_CBE	T2	8	0	0.00%	0/0
	T6	10	0	0.00%	0/0
rAPOBEC1(R33A)-CBE	T2	12	0	0.00%	0/0
	T6	11	0	0.00%	0/0

### Detection of off-target sites for A3A-CBE binary vector in transgenic tobacco plants

The off-target events for A3A-CBE binary vector in target sites T2 and T6 were evaluated using an online tool CRISPOR (http://crispor.tefor.net/). We identified nine potential off-target sites for A3A-CBE binary vector which were listed in Table 2. The genomic DNA from transgenic plants was used as a PCR template to amplify potential off-target sites and the PCR products were sent for sanger sequencing. We did not observe C to T conversion in all the potential off-target sites in transgenic plants (Table 2). These results showed that A3A-CBE binary vector specifically carried out C to T conversions.

**Table 2:** Analysis of off-target events induced by A3A-CBE at T2 and T6 target sites in transformed tobacco. Green bases represented mismatch bases

Target	Off-target sites	Putative off-target sequences	Chromosome	Start	End	The number of transgenic plants	Number of lines with off-targets
T2	1	GGTAGACAAAAAATAAAAGATGG	Niben101Ctg15924Ctg001	905	927	9	0
	2	TATAACTAAAACATAAAGAAGGG	Niben101Scf02262Ctg025	1087	1109	9	0
	3	AGGAAACAAAACATAAAGGAAGG	Niben101Scf08519Ctg012	728	750	9	0
	4	TTTAGTAAATACATAAAGGAGGG	Niben101Scf05619Ctg006	16911	16933	9	0
	5	TGTTGTCAATACATAAAGAAAGG	Niben101Scf03026Ctg060	23355	23377	9	0
T6	6	TAGGTTCTGAAAATTGAGTTTGG	Niben101Scf08162Ctg007	54182	54204	9	0
	7	AAATTATTGCAAATTGATTTTGG	Niben101Scf08857Ctg003	22514	22536	9	0
	8	GTACTACTGCAAATTAAGTTAGG	Niben101Scf09870Ctg011	8274	8296	9	0
	9	GAGTTGCAGCAAATTAAGTATGG	Niben101Scf00605Ctg019	2465	2487	9	0

## Discussion

Gene editing techniques have widely been used in the precise molecular breeding of crops, including *Oryza sativa*, *Triticum aestivum*, *Lycopersicon esculentum*, *Citrullus lanatus*, *Gossypium hirsutum*, *Glycine max*, and *Brassica napus* [20–26]. The base editors can significantly increase gene editing efficiency. In human cells, the DNA editing efficiency of rAPOBEC1(R33A) was similar to rAPOBEC1, while the RNA editing efficiency of rAPOBEC1(R33A) was lower than that of rAPOBEC1 [12]. A3A (Y130F) showed excellent editing ability in *Lycopersicon esculentum*, *Oryza sativa*, and *Arabidopsis thaliana* [13, 14]. However, all CBE binary vector showed a certain proportion of deletion and non-specific editing events, and recruitment of UGIs can reduce the occurrence of these events [7]. In current study, we constructed four CBE binary vectors (all vectors recruit two UGIs) to assess their efficiencies for converting C to T in the model plant *N. benthamiana* (Fig. 1A).

Previous study showed that the editing efficiencies of ABE using *slEF1α* promoter were significantly higher than using 35S promoter in tomato and soybean [27]. However, *slEF1ɑ*-A3A-CBE we constructed had not such effect, which might due to difference genomic background. Researchers have shown that base background significantly affected the editing efficiency of CBE binary vectors [11, 19]. The comparative study of the editing efficiency of three different base editors in tobacco under different base backgrounds showed that the two vectors [A3A-CBE and A3A (Y130F)-CBE] had the highest editing efficiency under the base background of CC in transgenic tobacco plants. This phenomenon was different from human cells, rice, and *Arabidopsis*, possibly because of different genetic make-up of tobacco. The base editing efficiency results of CBE binary vectors in stable transformation were consistent with those of transient transformation of tobacco plants, and both methods showed the highest editing efficiency for A3A-CBE. Interestingly, the CBE binary vectors failed to edit base pairs in target sites T4 and T13 during deep sequencing analysis, and the mechanism needed to be further analyzed. CBE induced substantial genome-wide off-target mutations in rice and various cells [28], but our results showed no off-target event, which might be due to the use of two UGIs. Previous study had reported similar result where CBE recruiting more UGIs improved the purity of the product [14]. A3A-CBE might find off-target events adoption on whole genome sequencing. In subsequent studies, the selection of new deaminase is an important direction to reduce off-target event, such as TadA derivative [29], rAPOBEC1 mutants [30] and truncated A3A [31].

According to the previous research results, the optimized codon can improve the efficiency of CRISPR/Cas9 and base editors [1]. Therefore, we think that the optimized codon of A3A-CBE binary vector can further enhance its editing efficiency in plant cells. A previous study showed that base editing could also be used to edit so-called domestication genes to accelerate the domestication of wild plants [32]. Previous studies have also shown that polycistronic tRNA-gRNA expression cassette (PTG) can improve the editing efficiency of CRISPR/Cas9 and base editors in polyploid species [33]. Hence, we believe that our proposed CBE binary vectors containing PTG will assist researcher to efficiently perform SNP-based plant breeding in polyploid plants.

## Conclusion

In the present study, we constructed three different CBE binary vectors containing different deaminases to assess their efficiency and accuracy for gene base editing. The experimental results showed that A3A-CBE binary vector was the most efficient CBE, and its high efficiency could be used in plant accurate molecular breeding and crop character improvement. The A3A-CBE binary vector will efficiently and accurately edit genes in SNPs-based plant breeding in polyploid plants.

## Methods

### Construction of CBE binary vectors

We used the JCat tool (http://www.jcat.de/) for codon optimization by adding linkers with the sequences of A3A, A3A(Y130F), and rAPOBEC1(R33A). The sequences of linker 1-A3A-linker 2, linker 1-A3A(Y130F)-linker 2, and linker 1-rAPOBEC1(R33A)-linker 2 were commercially synthesized by Genewiz from Azenta Life Sciences (Genewiz, Suzhou, China). The JCat tool (http://www.jcat.de/) was used to codon-optimized 2 × UGI-NLS sequences, and the sequences were commercially synthesized by Genewiz from Azenta Life Sciences (Genewiz, Suzhou, China). Firstly, the nCas9-NLS fragment was replaced with the EYFP segment between the BamHI and the SpeI restriction sites in the pGreen-EYFP-AtU6-26-DN vector to form pGreen-nCas9-DN. Then, the 2 × UGI-NLS was introduced into the pGreen-nCas9-DN vector to create the pGreen-nCas9-2 × UGI-DN. Finally, the A3A, A3A(Y130F), and rAPOBEC1(R33A), including the linkers, were inserted into the vector pGreen-nCas9-2 × UGI-DN to form the pGreen-A3A-nCas9-2 × UGI-DN (named as A3A-CBE), pGreen-A3A(Y130F)-nCas9-2 × UGI-DN, [named as A3A(Y130F)-CBE], and pGreen-rAPOBEC1(R33A)-nCas9-2 × UGI-DN [named as rAPOBEC1(R33A)-CBE], respectively. The detailed base sequences of these three CBE vectors shown in Figures S8, S9 and S10. Replaced the *CaMV* 35S promoter between the SnaBI and the XbaI restriction sites in A3A-CBE with *slEF1ɑ* promoter to get a new CBE, named *slEF1ɑ*-A3A-CBE (the detailed base sequences shown in Figure S11).

An online CRISPOR tool (http://crispor.tefor.net/) was employed to design the 14 sgRNAs targeting the *NbPDS* gene (Table S1). The pairs of primers for sgRNAs were created using an in-house Perl script and synthesized by TsingKe Biotech (TsingKe, Beijing, China) (Table S2). We cloned each of the sgRNAs into different CBE binary vectors by following a previously described protocol [34].

### Transient overexpression of CBE binary vector in *N. benthamiana*

The *N. benthamiana* plants for transient overexpression experiment were grown in a growth chamber at 26 ℃ temperature and 16 h photoperiod. The transformation in *N. benthamiana* was performed by following a previously described method [35]. The EHA105 host cells harboring the CBE binary vector were cultured on an LB medium supplemented with Kanamycin 50 mg L^−1^ and Streptomycin 25 mg L^−1^ at 28 ℃ temperature. The EHA105 host cells were then pelleted and resuspended in MES buffer to maintain the OD_600_ = 0.5. The Bacterial culture was placed under dark conditions for 2 ~ 3 h at room temperature (RT) before infiltration. Then, the 4 ~ 5 weeks old *N. benthamiana* leaves were infiltrated with the bacterial culture of the EHA105 strain harboring the CBE binary vectors. Finally, the leaves of *N. banthamiana* were harvested at 72 h post infiltration. The genomic DNA was extracted by an unclean plant genomic DNA commercial kit (CWBIO, Beijing, China) to identify base editing.

### Deep amplicon sequencing and data analysis

The genomic DNA extracted from transformed leaves of *N. benthamiana* was used as a template for PCR. The first step of PCR was carried out to amplify the targeted genomic region by specific pair of primers using the TransStart® FastPfu DNA Polymerase (TransGen Biotech, Beijing, China). Further, the forward and reverse barcodes were added to the first PCR products for library construction. The second step of PCR was carried out to attach adaptors to the amplicon. The Illumina Hiseq 2500 platform (Lc-Biotechnologies, Hangzhou, China) was used to perform amplicon sequencing. The clean read number for amplicon sequencing ranged between 54,000 ~ 88,000. All the experiments were repeated thrice. The on-target base editing efficiencies were analyzed using CRISPResso2 software with default parameters [17].

### *Agrobacterium*-mediated transformation of targeted genomic regions in *N. benthamiana*

The *N. benthamiana* plants were used for stable transformation of targeted genomic regions by following a previously described protocol [35]. The transformants were screened against a 100 mg L^*−*1^ kanamycin selection marker. The positive transgenic plants were used for the extraction of genomic DNA. The amplicons were amplified from genomic DNA by using specific pair of primers. The amplified amplicons were cloned into the pClone007 vector using the pClone007 simple vector kit (TsingKe, Beijing, China). The ligated products were transformed into *E. coli* strain DH5α cells and 30 positive colonies were selected for sanger sequencing. The sequencing results were verified using DNAMAN software v4.0 (Lynnon Corporation, Vaudreuil, Canada).

### Statistical analysis

All the statistical analyses were carried out on Graphpad Prism 9. The results were subjected to t-test. The mean differences were presented as mean ± SE. All the experiments in this study were repeated three times.

## Supplementary Information

**Additional file 1:** **Figure S1.** The editing efficiencies of A3A-CBE, A3A (Y130F)-CBE, and rAPOBEC1(R33A)-CBE for converting C-to-T at (A) T1 ~ T7 target sites, and (B) T8 ~ T14 target sites. The editing efficiencies were calculated from three independent replicates' deep sequencing analysis results.

**Additional file 2:** **Figure S2. **The editing efficiencies of A3A-CBE, A3A (Y130F)-CBE, and rAPOBEC1(R33A)-CBE for converting C-to-G at (A) T1 ~ T7 target sites, and (B) T8 ~ T14 target sites. The editing efficiencies were calculated from three independent replicates' deep sequencing analysis results.

**Additional file 3:** **Figure S3. **The editing efficiencies of A3A-CBE, A3A (Y130F)-CBE, and rAPOBEC1(R33A)-CBE for converting C-to-A at (A) T1 ~ T7 target sites, and (B) T8 ~ T14 target sites. The editing efficiencies were calculated from three independent replicates' deep sequencing analysis results.

**Additional file 4:** **Figure S4. **The indel frequencies of A3A-CBE, A3A (Y130F)-CBE, and rAPOBEC1(R33A)-CBE for converting C-to-delete at (A) T1 ~ T7 target sites, and (B) T8 ~ T14 target sites. The editing frequencies were calculated from three independent replicates' deep sequencing analysis results.

**Additional file 5:** **Figure S5. **The editing efficiencies of A3A-CBE, A3A (Y130F)-CBE, and rAPOBEC1(R33A)-CBE for converting C-to-N at (A) T1 ~ T7 target sites, and (B) T8 ~ T14 target sites. The editing efficiencies were calculated from three independent replicates' deep sequencing analysis results.

**Additional file 6:** **Figure S6. **Editing efficiency estimation of binary vectors A3A-CBE and *slEF1α*-A3A-CBE for converting C-to-T. (A) The schematic diagrams of A3A-CBE, and *slEF1α*-A3A-CBE. The detail base sequences of the *slEF1α*-A3A-CBE were shown in Figure S11. (B) Sanger sequencing peak map of the T1 target site, and the red boxes represent the edited base induced by CBEs. (C) The editing efficiencies were calculated from three independent replicates' sanger sequencing analysis results.

**Additional file 7:** **Figure S7. **The editing efficiencies of A3A-CBE, A3A (Y130F)-CBE, and rAPOBEC1(R33A)-CBE for converting C-to-T at C_1_ ~ C_20_ sites (except C_2_ site) of different NC motifs based on the results of deep sequencing analysis.

**Additional file 8:** **Figure S8. **The sequence of the A3A-CBE editing vector. Different colors represented different elements.

**Additional file 9:** **Figure S9. **The sequence of A3A(Y130F)-CBE editing vector. Different colors represented different elements.

**Additional file 10:** **Figure S10. **The sequence of the rAPOBEC1(R33A)-CBE editing vector. Different colors represented different elements.

**Additional file 11:** **Figure S11. **The sequence of the *slEF1α*-A3A-CBE editing vector. Different colors represented different elements.

**Additional file 12:** **Table S1. **The target sequences (T1 ~ T14) used in this study. Red letters indicated the PAM sequence of sgRNA.

**Additional file 13:** **Table S2. **Detail for all pairs of primers used in this study.

## Data Availability

The datasets used during the current study are available at https://solgenomics.net/organism/Nicotiana_benthamiana/genome. The datasets generated and analyzed during the current study are available in the NCBI Sequence Read Archive repository (BioProject Accession: PRJNA967404). The addresses are as follows: https://www.ncbi.nlm.nih.gov/sra/PRJNA967404.

## References

[1] Chen K, Wang Y, Zhang R, Zhang H, Gao C (2019). CRISPR/Cas Genome Editing and Precision Plant Breeding in Agriculture. Annu Rev Plant Biol.

[2] Henikoff S, Comai L (2003). Single-nucleotide mutations for plant functional genomics. Annu Rev Plant Biol.

[3] Li C, Zhang R, Meng X, Chen S, Zong Y, Lu C, Qiu JL, Chen YH, Li J, Gao C (2020). Targeted, random mutagenesis of plant genes with dual cytosine and adenine base editors. Nat Biotechnol.

[4] Kuang Y, Li S, Ren B, Yan F, Spetz C, Li X, Zhou X, Zhou H (2020). Base-Editing-Mediated Artificial Evolution of OsALS1 In Planta to Develop Novel Herbicide-Tolerant Rice Germplasms. Mol Plant.

[5] Salsman J, Dellaire G (2017). Precision genome editing in the CRISPR era. Biochem Cell Biol..

[6] Bharat SS, Li S, Li J, Yan L, Xia L (2020). Base editing in plants: Current status and challenges. The Crop Journal.

[7] Azameti MK, Dauda WP (2021). Base Editing in Plants: Applications, Challenges, and Future Prospects. Front Plant Sci.

[8] Anzalone AV, Koblan LW, Liu DR (2020). Genome editing with CRISPR-Cas nucleases, base editors, transposases and prime editors. Nat Biotechnol.

[9] Gaudelli NM, Komor AC, Rees HA, Packer MS, Badran AH, Bryson DI, Liu DR (2017). Programmable base editing of A*T to G*C in genomic DNA without DNA cleavage. Nature.

[10] Kurt IC, Zhou R, Iyer S, Garcia SP, Miller BR, Langner LM, Grunewald J, Joung JK (2021). CRISPR C-to-G base editors for inducing targeted DNA transversions in human cells. Nat Biotechnol.

[11] Komor AC, Kim YB, Packer MS, Zuris JA, Liu DR (2016). Programmable editing of a target base in genomic DNA without double-stranded DNA cleavage. Nature.

[12] Grunewald J, Zhou R, Garcia SP, Iyer S, Lareau CA, Aryee MJ, Joung JK (2019). Transcriptome-wide off-target RNA editing induced by CRISPR-guided DNA base editors. Nature.

[13] Randall LB, Sretenovic S, Wu Y, Yin D, Zhang T, Eck JV, Qi Y (2021). Genome- and transcriptome-wide off-target analyses of an improved cytosine base editor. Plant Physiol.

[14] Ren Q, Sretenovic S, Liu G, Zhong Z, Wang J, Huang L, Tang X, Guo Y, Liu L, Wu Y (2021). Improved plant cytosine base editors with high editing activity, purity, and specificity. Plant Biotechnol J.

[15] Zong Y, Song Q, Li C, Jin S, Zhang D, Wang Y, Qiu JL, Gao C (2018). Efficient C-to-T base editing in plants using a fusion of nCas9 and human APOBEC3A. Nat Biotechnol..

[16] Kluesner MG, Nedveck DA, Lahr WS, Garbe JR, Abrahante JE, Webber BR, Moriarity BS (2018). EditR: A Method to Quantify Base Editing from Sanger Sequencing. CRISPR J.

[17] Clement K, Rees H, Canver MC, Gehrke JM, Farouni R, Hsu JY, Cole MA, Liu DR, Joung JK, Bauer DE (2019). CRISPResso2 provides accurate and rapid genome editing sequence analysis. Nat Biotechnol.

[18] Song M, Kim HK, Lee S, Kim Y, Seo SY, Park J, Choi JW, Jang H, Shin JH, Min S (2020). Sequence-specific prediction of the efficiencies of adenine and cytosine base editors. Nat Biotechnol.

[19] Ren B, Yan F, Kuang Y, Li N, Zhang D, Lin H, Zhou H (2017). A CRISPR/Cas9 toolkit for efficient targeted base editing to induce genetic variations in rice. Sci China Life Sci.

[20] Hua K, Jiang Y, Tao X, Zhu JK (2020). Precision genome engineering in rice using prime editing system. Plant Biotechnol J.

[21] Zhang R, Liu J, Chai Z, Chen S, Bai Y, Zong Y, Chen K, Li J, Jiang L, Gao C (2019). Generation of herbicide tolerance traits and a new selectable marker in wheat using base editing. Nat Plants.

[22] Veillet F, Perrot L, Chauvin L, Kermarrec MP, Guyon-Debast A, Chauvin JE, Nogue F, Mazier M (2019). Transgene-Free Genome Editing in Tomato and Potato Plants Using Agrobacterium-Mediated Delivery of a CRISPR/Cas9 Cytidine Base Editor. Int J Mol Sci.

[23] Tian S, Jiang L, Cui X, Zhang J, Guo S, Li M, Zhang H, Ren Y, Gong G, Zong M (2018). Engineering herbicide-resistant watermelon variety through CRISPR/Cas9-mediated base-editing. Plant Cell Rep.

[24] Qin L, Li J, Wang Q, Xu Z, Sun L, Alariqi M, Manghwar H, Wang G, Li B, Ding X (2020). High-efficient and precise base editing of C*G to T*A in the allotetraploid cotton (Gossypium hirsutum) genome using a modified CRISPR/Cas9 system. Plant Biotechnol J.

[25] Cai Y, Chen L, Zhang Y, Yuan S, Su Q, Sun S, Wu C, Yao W, Han T, Hou W (2020). Target base editing in soybean using a modified CRISPR/Cas9 system. Plant Biotechnol J..

[26] Wu J, Chen C, Xian G, Liu D, Lin L, Yin S, Sun Q, Fang Y, Zhang H, Wang Y (2020). Engineering herbicide-resistant oilseed rape by CRISPR/Cas9-mediated cytosine base-editing. Plant Biotechnol J.

[27] Niu Q, Wu S, Xie H, Wu Q, Liu P, Xu Y, Lang Z (2022). Efficient A·T to G·C base conversions in dicots using adenine base editors expressed under the tomato EF1α promoter. Plant Biotechnol J.

[28] Jin S, Zong Y, Gao Q, Zhu Z, Wang Y, Qin P, Liang C, Wang D, Qiu J-L, Zhang F (2019). Cytosine, but not adenine, base editors induce genome-wide off-target mutations in rice. Science.

[29] Neugebauer ME, Hsu A, Arbab M, Krasnow NA, McElroy AN, Pandey S, Doman JL, Huang TP, Raguram A, Banskota S (2022). Evolution of an adenine base editor into a small, efficient cytosine base editor with low off-target activity. Nat Biotechnol..

[30] Zuo E, Sun Y, Yuan T, He B, Zhou C, Ying W, Liu J, Wei W, Zeng R, Li Y (2020). A rationally engineered cytosine base editor retains high on-target activity while reducing both DNA and RNA off-target effects. Nat Methods.

[31] Jin S, Fei H, Zhu Z, Luo Y, Liu J, Gao S, Zhang F, Chen YH, Wang Y, Gao C (2020). Rationally Designed APOBEC3B Cytosine Base Editors with Improved Specificity. Mol Cell.

[32] Osterberg JT, Xiang W, Olsen LI, Edenbrandt AK, Vedel SE, Christiansen A, Landes X, Andersen MM, Pagh P, Sandoe P (2017). Accelerating the Domestication of New Crops: Feasibility and Approaches. Trends Plant Sci.

[33] Chon C, Chon G, Matsui Y, Zeng H, Lai ZC, Liu A (2021). Efficient multiplexed genome engineering with a polycistronic tRNA and CRISPR guide-RNA reveals an important role of detonator in reproduction of Drosophila melanogaster. PLoS ONE.

[34] Wang Z, Wang S, Li D, Zhang Q, Li L, Zhong C, Liu Y, Huang H (2018). Optimized paired-sgRNA/Cas9 cloning and expression cassette triggers high-efficiency multiplex genome editing in kiwifruit. Plant Biotechnol J.

[35] Shan Q, Wang Y, Li J, Zhang Y, Chen K, Liang Z, Zhang K, Liu J, Xi JJ, Qiu J-L (2013). Targeted genome modification of crop plants using a CRISPR-Cas system. Nat Biotechnol.

