# Supplementary figures and images for: Cytosine base editors (CBEs) for inducing targeted DNA base editing in Nicotiana benthamiana
Source: BMC Plant Biol. 2023 Jun 7;23:305. doi: 10.1186/s12870-023-04322-8 (PMC10245509; doi:10.1186/s12870-023-04322-8)

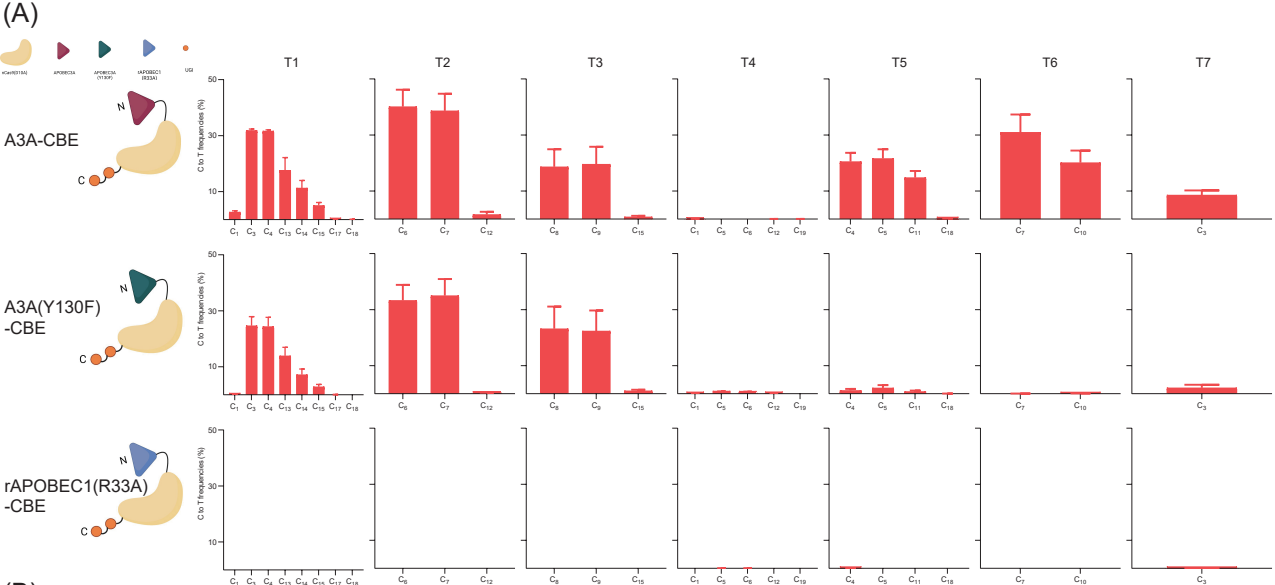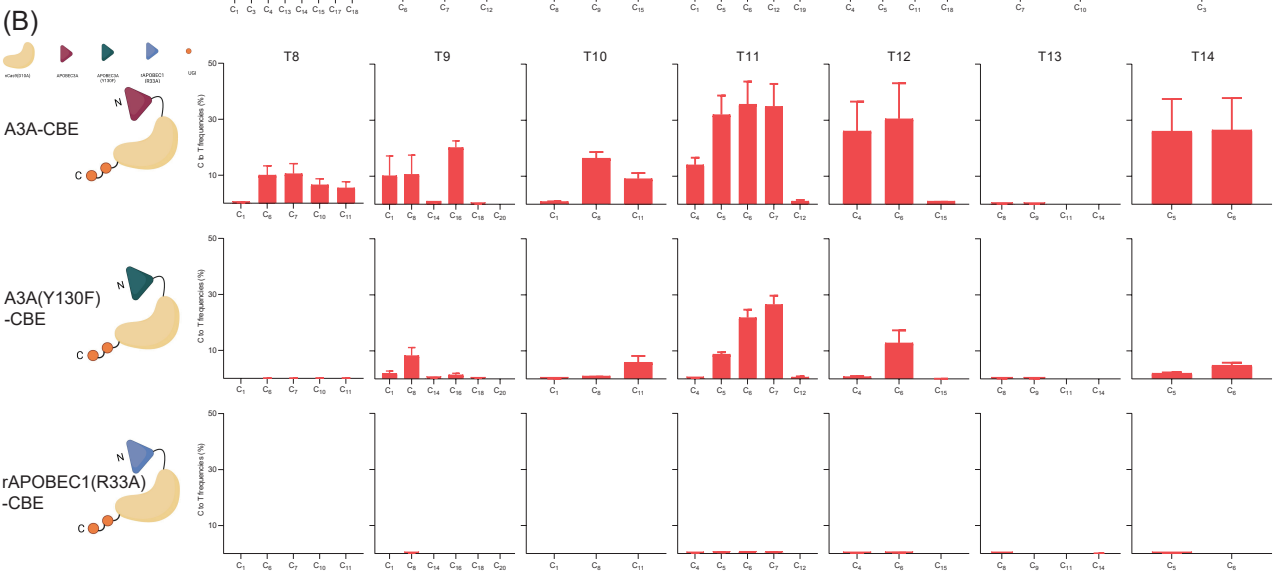

Supplement: Supplementary file 1 — Additional file 1: Figure S1. The editing efficiencies of A3A-CBE, A3A (Y130F)-CBE, and rAPOBEC1(R33A)-CBE for converting C-to-T at (A) T1 ~ T7 target sites, and (B) T8 ~ T14 target sites. The editing efficiencies were calculated from three independent replicates' deep sequencing analysis results. [file 12870_2023_4322_MOESM1_ESM.pdf]

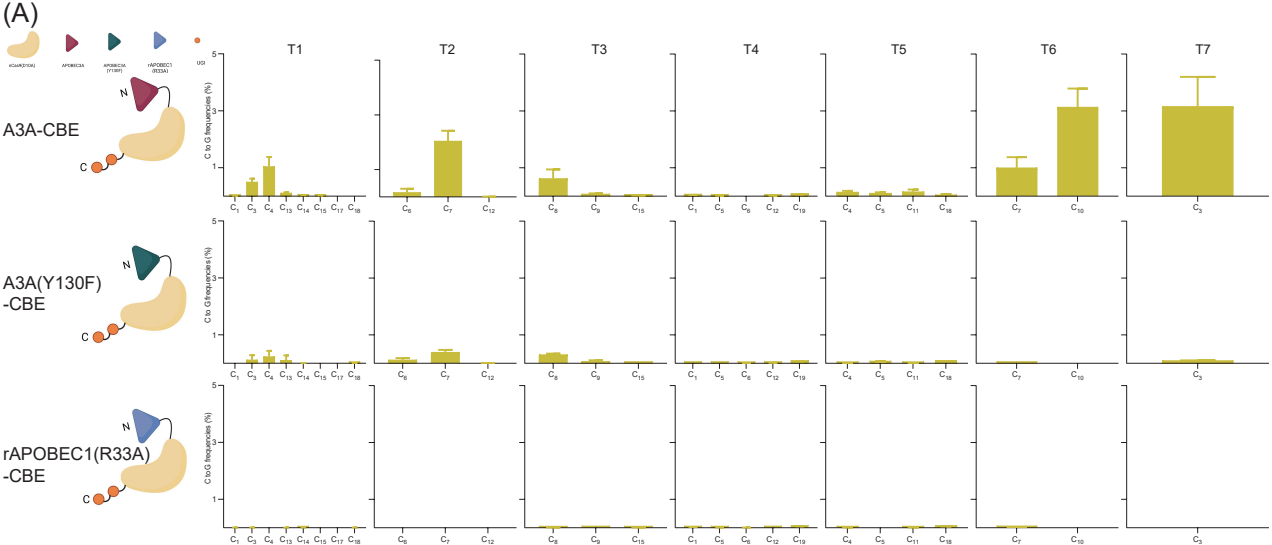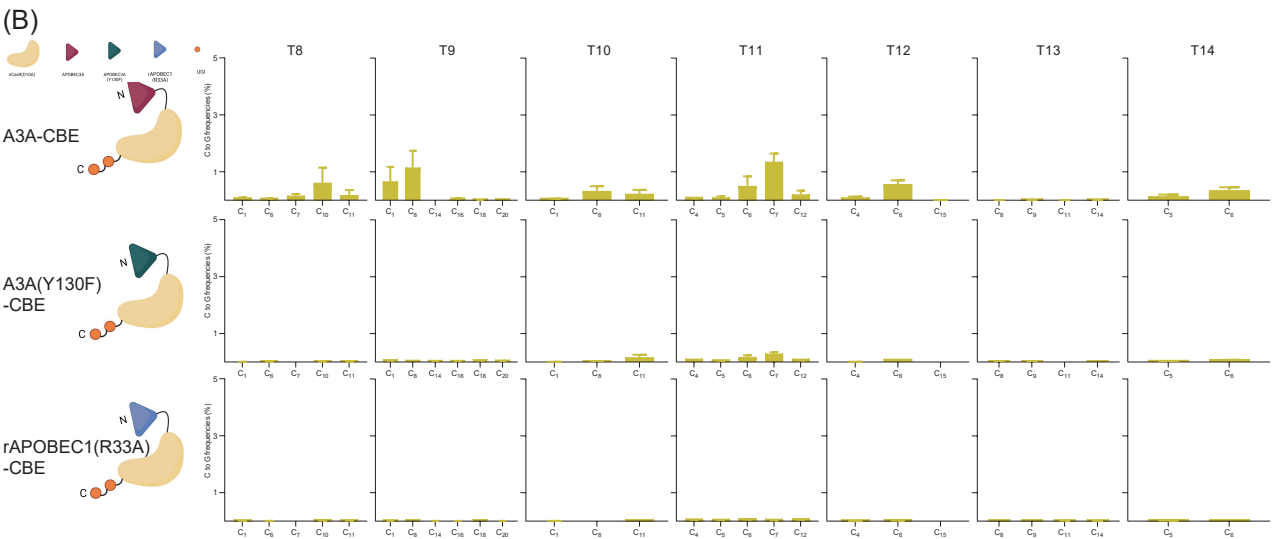

Supplement: Supplementary file 2 — Additional file 2: Figure S2. The editing efficiencies of A3A-CBE, A3A (Y130F)-CBE, and rAPOBEC1(R33A)-CBE for converting C-to-G at (A) T1 ~ T7 target sites, and (B) T8 ~ T14 target sites. The editing efficiencies were calculated from three independent replicates' deep sequencing analysis results. [file 12870_2023_4322_MOESM2_ESM.pdf]

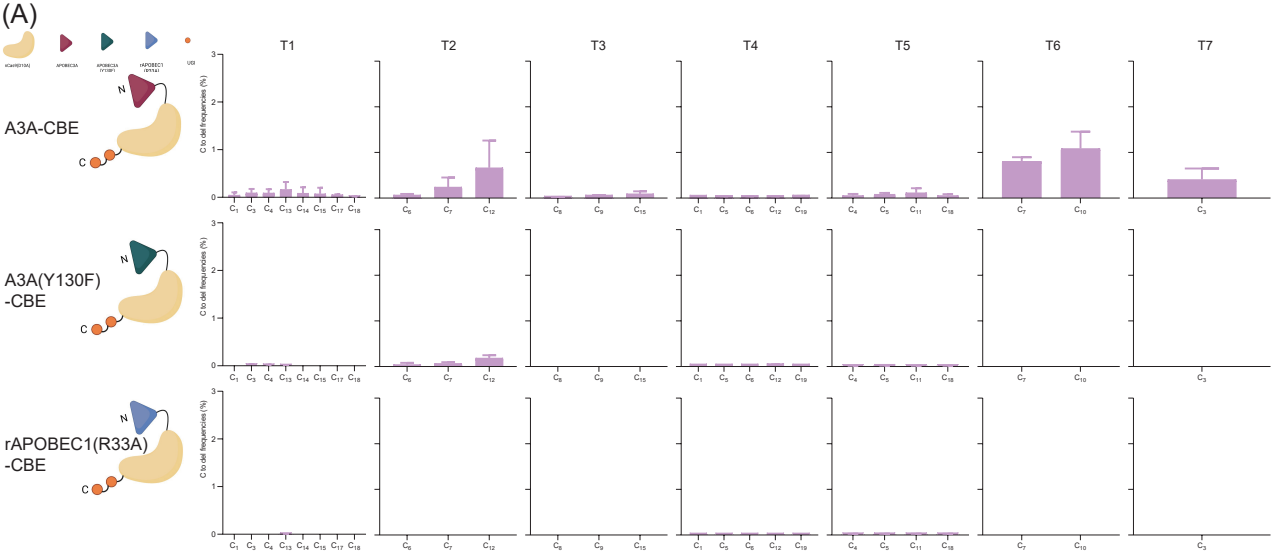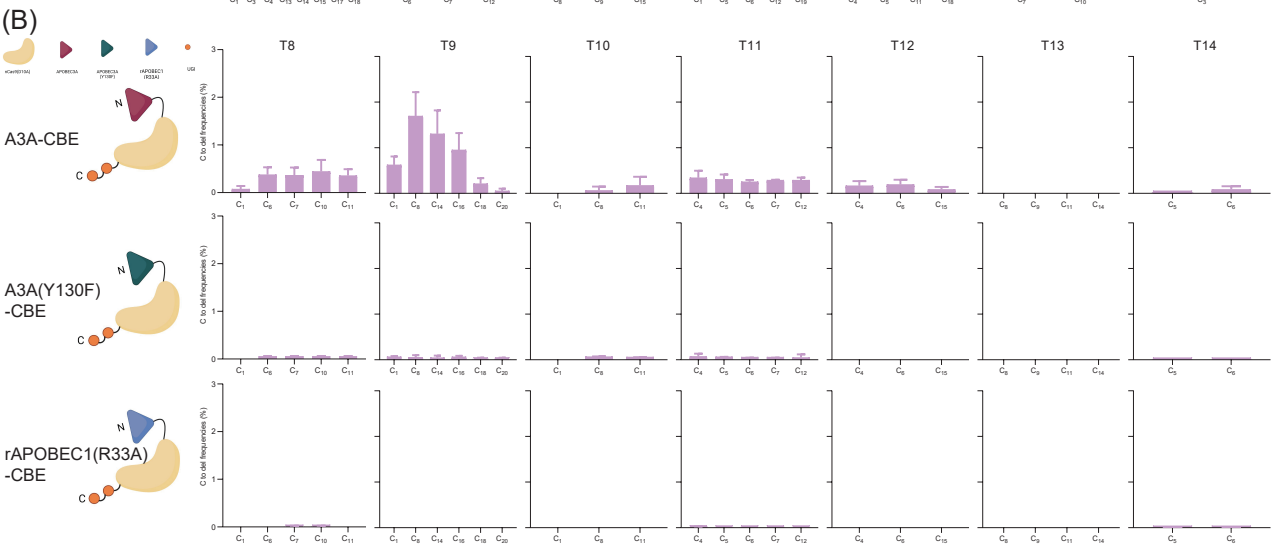

Supplement: Supplementary file 4 — Additional file 4: Figure S4. The indel frequencies of A3A-CBE, A3A (Y130F)-CBE, and rAPOBEC1(R33A)-CBE for converting C-to-delete at (A) T1 ~ T7 target sites, and (B) T8 ~ T14 target sites. The editing frequencies were calculated from three independent replicates' deep sequencing analysis results. [file 12870_2023_4322_MOESM4_ESM.pdf]

(A)

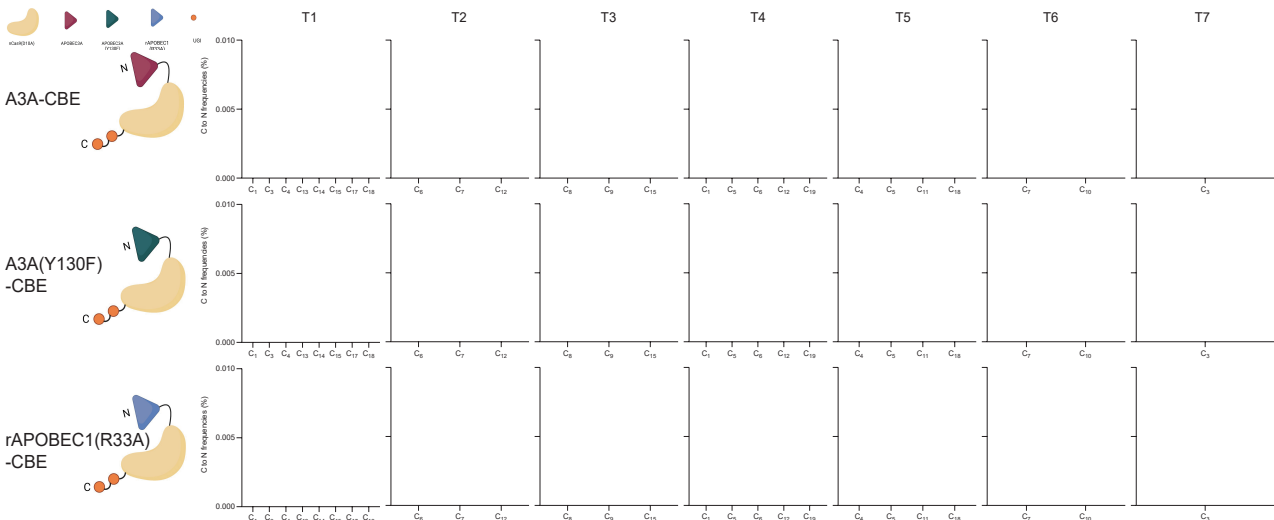

(B)

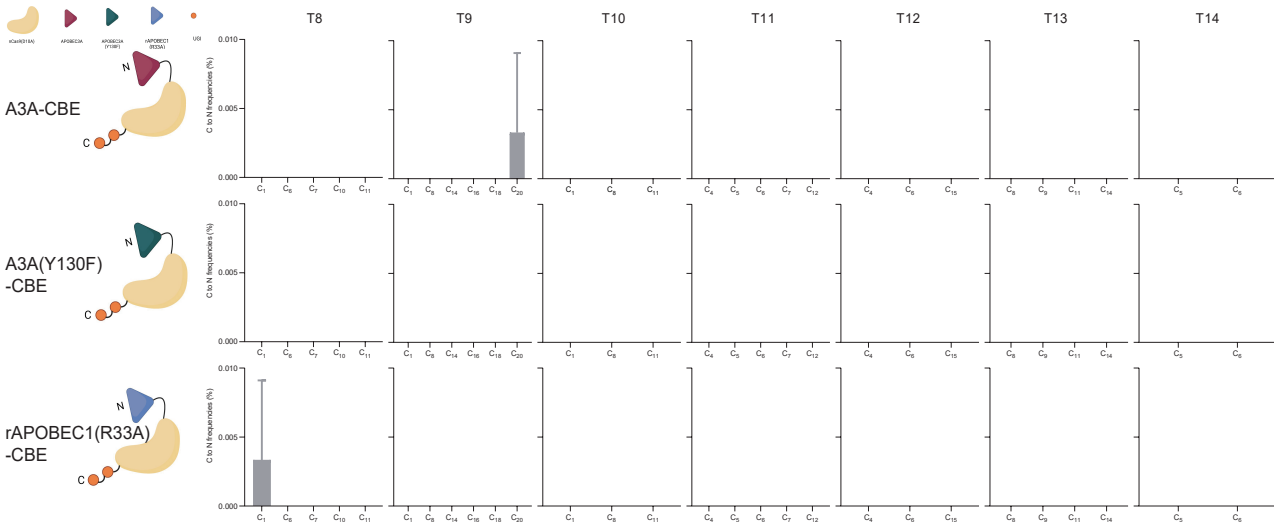

Supplement: Supplementary file 5 — Additional file 5: Figure S5. The editing efficiencies of A3A-CBE, A3A (Y130F)-CBE, and rAPOBEC1(R33A)-CBE for converting C-to-N at (A) T1 ~ T7 target sites, and (B) T8 ~ T14 target sites. The editing efficiencies were calculated from three independent replicates' deep sequencing analysis results. [file 12870_2023_4322_MOESM5_ESM.pdf]

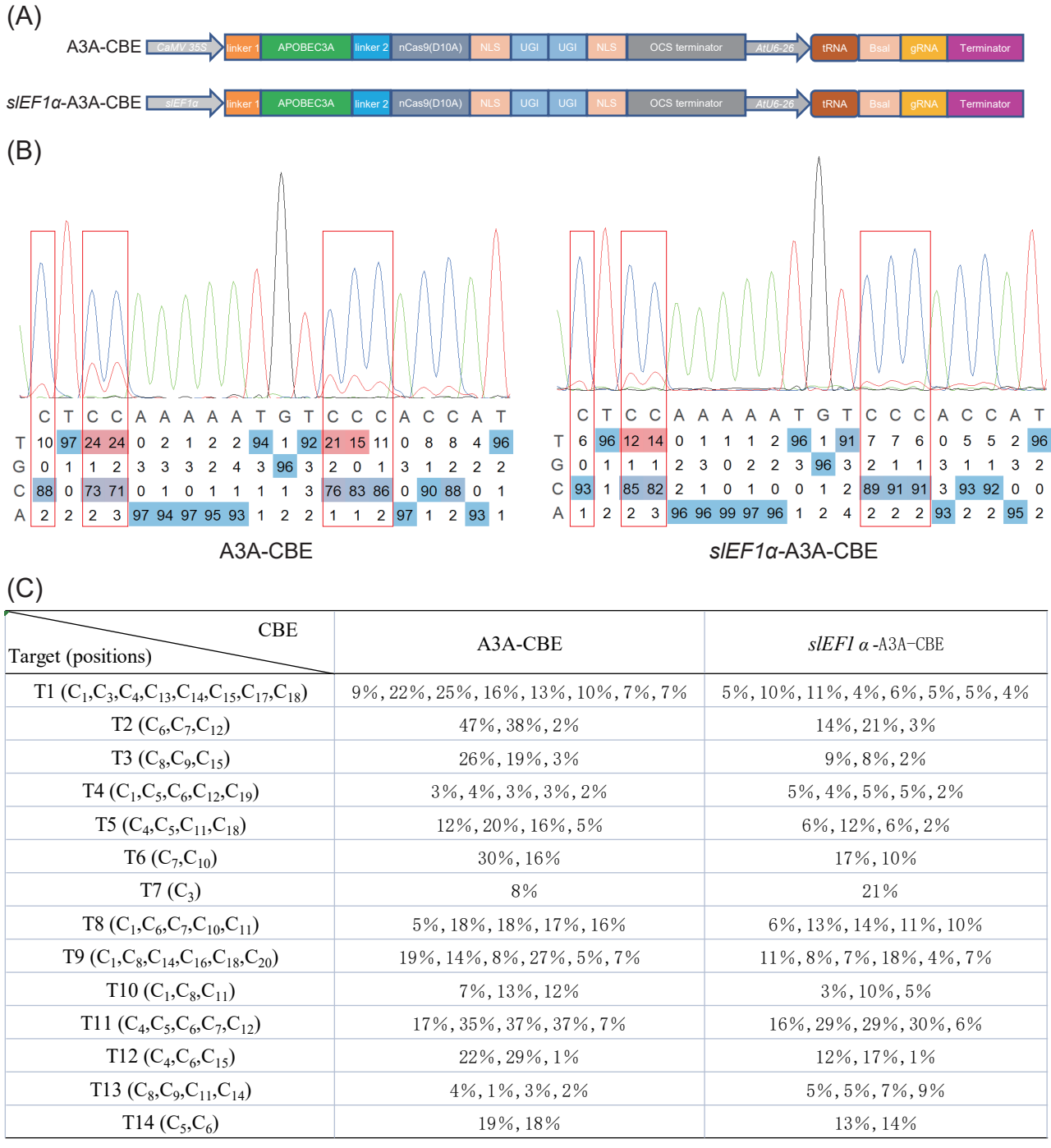

Supplement: Supplementary file 6 — Additional file 6: Figure S6. Editing efficiency estimation of binary vectors A3A-CBE and slEF1α-A3A-CBE for converting C-to-T. (A) The schematic diagrams of A3A-CBE, and slEF1α-A3A-CBE. The detail base sequences of the slEF1α-A3A-CBE were shown in Figure S11. (B) Sanger sequencing peak map of the T1 target site, and the red boxes represent the edited base induced by CBEs. (C) The editing efficiencies were calculated from three independent replicates' sanger sequencing analysis results. [file 12870_2023_4322_MOESM6_ESM.pdf]

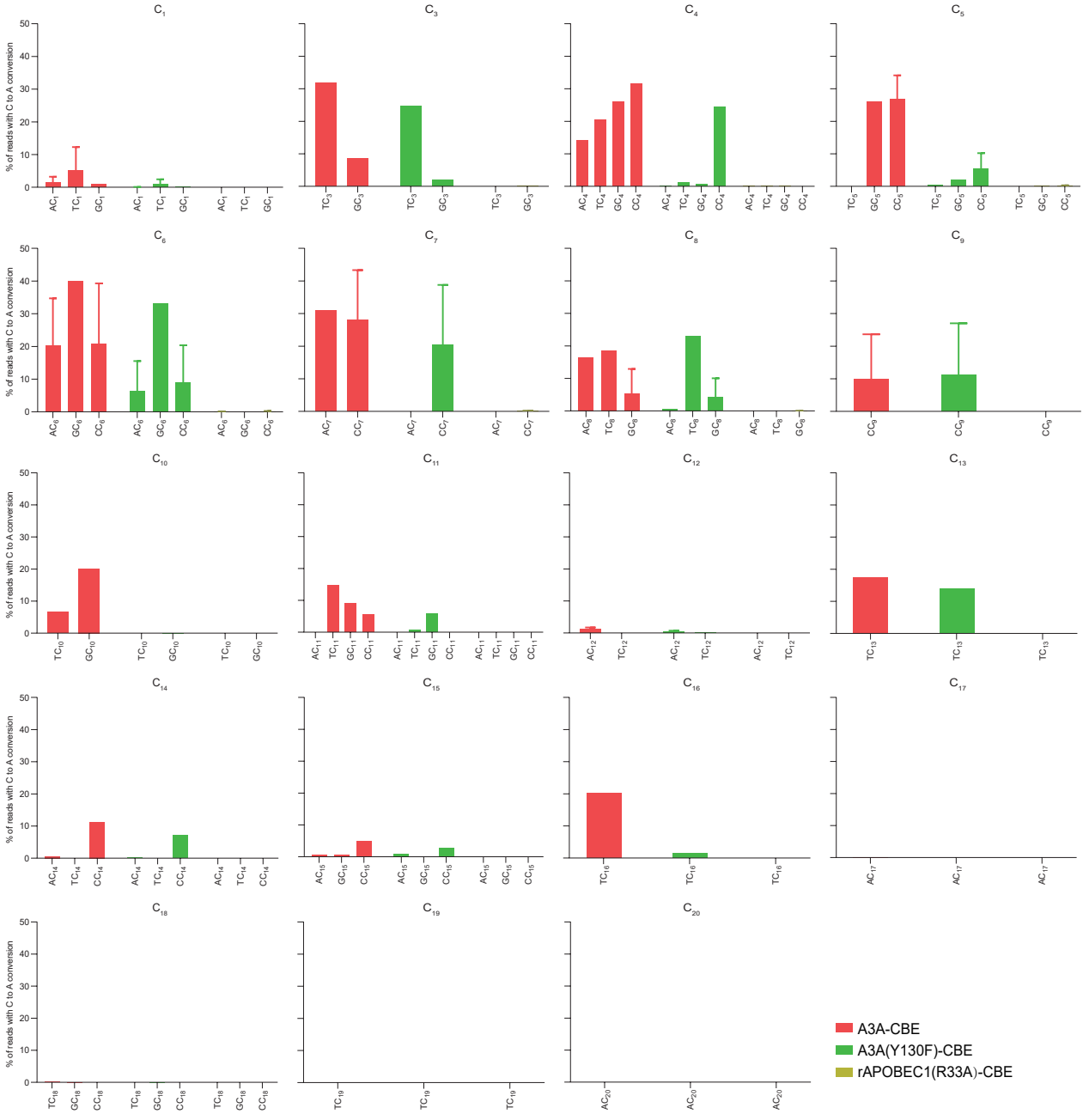

Supplement: Supplementary file 7 — Additional file 7: Figure S7. The editing efficiencies of A3A-CBE, A3A (Y130F)-CBE, and rAPOBEC1(R33A)-CBE for converting C-to-T at C1 ~ C20 sites (except C2 site) of different NC motifs based on the results of deep sequencing analysis. [file 12870_2023_4322_MOESM7_ESM.pdf]
