# Supplementary material for: Cytosine base editors (CBEs) for inducing targeted DNA base editing in Nicotiana benthamiana
Source: BMC Plant Biol. 2023 Jun 7;23:305. doi: 10.1186/s12870-023-04322-8 (PMC10245509; doi:10.1186/s12870-023-04322-8)
Supplement: Supplementary file 9 — Additional file 9: Figure S9. The sequence of A3A(Y130F)-CBE editing vector. Different colors represented different elements. [file 12870_2023_4322_MOESM9_ESM.pdf]

>A3A(Y130F)-CBE (35S promoter - linker 1 - A3A(Y130F) - linker 2 - nCas9- NLS - 2×UGI - NLS - OCS terminator - AtU6-26 - tRNA - BsaI - gRNA - Terminator)

tgagacttttcaacaaagggtaatttcgggaaacctcctcggattccattgcccagctatctgtcacttcatcgaaaggacagtagaaaaggaag
gtggctcctacaaatgccatcattgcgataaaggaaaggctatcattcaagatgcctctgccgacagtggtcccaaagatggacccccaccca
cgaggagcatcgtggaaaaagaagacgttccaaccacgtcttcaaagcaagtggattgatgtgacatctccactgacgtaagggatgacgca
caatcccactatccttcgcaagacccttcctctatataaggaagttcatttcatttggagaggacagcccaagctgagctccaccgcggtggcg
gccgctctagaactagacaattaccaacaacaacaaacaacaaacaacattacaattacatttacaattacggatccATGGAAGCTTC
TCCTGCTTCTGGACCTAGACATTTGATGGATCCTCATATTTTTACTTCTAATTTTAATAAT
GGAATTGGAAGACATAAGACTTATTTGTGTTATGAAGTTGAAAGATTGGATAATGGAAC
TTCTGTTAAGATGGATCAACATAGAGGATTTTTGCATAATCAAGCTAAGAATTTGTTGTG
TGGATTTTATGGAAGACATGCTGAATTGAGATTTTTGGATTTGGTTCCTTCTTTGCAATT
GGATCCTGCTCAAATTTATAGAGTTACTTGGTTTATTTCTTGGTCaCCTTGTTTTTCTTGG
GGATGTGCTGGAGAAGTTAGAGCTTTTTTGCAAGAAAATACTCATGTTAGATTGAGAAT
TTTTGCTGCTAGAATTTTTGATTATGATCCTTTGTATAAGGAAGCTTTGCAAATGTTGAG
AGATGCTGGAGCTCAAGTTTCTATTATGACTTATGATGAATTTAAGCATTGTTGGGATAC
TTTTGTTGATCATCAAGGATGTCCTTTTCAACCTTGGGATGGATTGGATGAACATTCTCA
AGCTTTGTCTGGAAGATTGAGAGCTATTTTGCAAAATCAAGGAAATTCTGGATCTGAA
ACTCCTGGAACTTCTGAATCTGCTACTCCTGAATCTCTGCAGGGATCCGACAAGAAGT
ACTCCATCGGCCTCGCCATCGGCACCAACAGCGTCGGCTGGGCGGTGATCACCGACGA
GTACAAGGTCCCGTCCAAGAAGTTCAAGGTCCTGGGCAACACCGACCGCCACTCCATC
AAGAAGAACCTCATCGGCGCCCTCCTCTTCGACTCCGGCGAGACGGCGGAGGCGACC
CGCCTCAAGCGCACCGCCCGCCGCCGCTACACCCGCCGCAAGAACCGCATCTGCTACC
TCCAGGAGATCTTCTCCAACGAGATGGCGAAGGTCGACGACTCCTTCTTCCACCGCCT
CGAGGAGTCCTTCCTCGTGGAGGAGGACAAGAAGCACGAGCGCCACCCCATCTTCGG
CAACATCGTCGACGAGGTCGCCTACCACGAGAAGTACCCCACTATCTACCACCTTCGTA
AGAAGCTTGTTGACTCTACTGATAAGGCTGATCTTCGTCTCATCTACCTTGCTCTCGCTC
ACATGATCAAGTTCCGTGGTCACTTCCTTATCGAGGGTGACCTTAACCCTGATAACTCC
GACGTGGACAAGCTCTTCATCCAGCTCGTCCAGACCTACAACCAGCTCTTCGAGGAGA
ACCCTATCAACGCTTCCGGTGTCGACGCTAAGGCGATCCTTTCCGCTAGGCTCTCCAAG
TCCAGGCGTCTCGAGAACCTCATCGCCCAGCTCCCTGGTGAGAAGAAGAACGGTCTTT
TCGGTAACCTCATCGCTCTCTCCCTCGGTCTGACCCCTAACTTCAAGTCCAACTTCGAC
CTCGCTGAGGACGCTAAGCTTCAGCTCTCCAAGGATACCTACGACGATGATCTCGACA
ACCTCCTCGCTCAGATTGGAGATCAGTACGCTGATCTCTTCCTTGCTGCTAAGAACCTC
TCCGATGCTATCCTCCTTTCGGATATCCTTAGGGTTAACACTGAGATCACTAAGGCTCCT
CTTTCTGCTTCCATGATCAAGCGCTACGACGAGCACCACCAGGACCTCACCCTCCTCA
AGGCTCTTGTTCGTCAGCAGCTCCCCGAGAAGTACAAGGAGATCTTCTTCGACCAGTC
CAAGAACGGCTACGCCGGTTACATTGACGGTGGAGCTAGCCAGGAGGAGTTCTACAA
GTTCATCAAGCCAATCCTTGAGAAGATGGATGGTACTGAGGAGCTTCTCGTTAAGCTTA
ACCGTGAGGACCTCCTTAGGAAGCAGAGGACTTTCGATAACGGCTCTATCCCTCACCA
GATCCACCTTGGTGAGCTTCACGCCATCCTTCGTAGGCAGGAGGACTTCTACCCTTTCC
TCAAGGACAACCGTGAGAAGATCGAGAAGATCCTTACTTTCCGTATTCCTTACTACGTT
GGTCCTCTTGCTCGTGGTAACTCCCGTTTCGCTTGGATGACTAGGAAGTCCGAGGAGA
CTATCACCCCTTGGAACTTCGAGGAGGTTGTTGACAAGGGTGCTTCCGCCCAGTCCTT
CATCGAGCGCATGACCAACTTCGACAAGAACCTCCCCAACGAGAAGGTCCTCCCCAA

GCACTCCCTCCTCTACGAGTACTTCACGGTCTACAACGAGCTCACCAAGGTCAAGTAC
GTCACCGAGGGTATGCGCAAGCCTGCCTTCCTCTCCGGCGAGCAGAAGAAGGCTATCG
TTGACCTCCTCTTCAAGACCAACCGCAAGGTCACCGTCAAGCAGCTCAAGGAGGACT
ACTTCAAGAAGATCGAGTGCTTCGACTCCGTCGAGATCAGCGGCGTTGAGGACCGTTT
CAACGCTTCTCTCGGTACCTACCACGATCTCCTCAAGATCATCAAGGACAAGGACTTCC
TCGACAACGAGGAGAACGAGGACATCCTCGAGGACATCGTCCTCACTCTTACTCTCTT
CGAGGATAGGGAGATGATCGAGGAGAGGCTCAAGACTTACGCTCATCTCTTCGATGAC
AAGGTTATGAAGCAGCTCAAGCGTCGCCGTTACACCGGTTGGGGTAGGCTCTCCCGCA
AGCTCATCAACGGTATCAGGGATAAGCAGAGCGGCAAGACTATCCTCGACTTCCTCAA
GTCTGATGGTTTCGCTAACAGGAACTTCATGCAGCTCATCCACGATGACTCTCTTACCT
TCAAGGAGGATATTCAGAAGGCTCAGGTGTCCGGTCAGGGCGACTCTCTCCACGAGCA
CATTGCTAACCTTGCTGGTTCCCCTGCTATCAAGAAGGGCATCCTTCAGACTGTTAAGG
TTGTCGATGAGCTTGTCAAGGTTATGGGTCGTCACAAGCCTGAGAACATCGTCATCGA
GATGGCTCGTGAGAACCAGACTACCCAGAAGGGTCAGAAGAACTCGAGGGAGCGCAT
GAAGAGGATTGAGGAGGGTATCAAGGAGCTTGGTTCTCAGATCCTTAAGGAGCACCCT
GTCGAGAACACCCAGCTCCAGAACGAGAAGCTCTACCTCTACTACCTCCAGAACGGTA
GGGATATGTACGTTGACCAGGAGCTCGACATCAACAGGCTTTCTGACTACGACGTCGA
CCACATTGTTCCTCAGTCTTTCCTTAAGGATGACTCCATCGACAACAAGGTCCTCACGA
GGTCCGACAAGAACAGGGGTAAGTCGGACAACGTCCCTTCCGAGGAGGTTGTCAAGA
AGATGAAGAACTACTGGAGGCAGCTTCTCAACGCTAAGCTCATTACCCAGAGGAAGTT
CGACAACCTCACGAAGGCTGAGAGGGGTGGCCTTTCCGAGCTTGACAAGGCTGGTTT
CATCAAGAGGCAGCTTGTTGAGACGAGGCAGATTACCAAGCACGTTGCTCAGATCCTC
GATTCTAGGATGAACACCAAGTACGACGAGAACGACAAGCTCATCCGCGAGGTCAAG
GTGATCACCCTCAAGTCCAAGCTCGTCTCCGACTTCCGCAAGGACTTCCAGTTCTACA
AGGTCCGCGAGATCAACAACTACCACCACGCTCACGATGCTTACCTTAACGCTGTCGT
TGGTACCGCTCTTATCAAGAAGTACCCTAAGCTTGAGTCCGAGTTCGTCTACGGTGACT
ACAAGGTCTACGACGTTCGTAAGATGATCGCCAAGTCCGAGCAGGAGATCGGCAAGG
CCACCGCCAAGTACTTCTTCTACTCCAACATCATGAACTTCTTCAAGACCGAGATCACC
CTCGCCAACGGCGAGATCCGCAAGCGCCCTCTTATCGAGACGAACGGTGAGACTGGT
GAGATCGTTTGGGACAAGGGTCGCGACTTCGCTACTGTTCGCAAGGTCCTTTCTATGCC
TCAGGTTAACATCGTCAAGAAGACCGAGGTCCAGACCGGTGGCTTCTCCAAGGAGTCT
ATCCTTCCAAAGAGAAACTCGGACAAGCTCATCGCTAGGAAGAAGGATTGGGACCCTA
AGAAGTACGGTGGTTTCGACTCCCCTACTGTCGCCTACTCCGTCCTCGTGGTCGCCAA
GGTGGAGAAGGGTAAGTCGAAGAAGCTCAAGTCCGTCAAGGAGCTCCTCGGCATCAC
CATCATGGAGCGCTCCTCCTTCGAGAAGAACCCGATCGACTTCCTCGAGGCCAAGGGC
TACAAGGAGGTCAAGAAGGACCTCATCATCAAGCTCCCCAAGTACTCTCTTTTCGAGC
TCGAGAACGGTCGTAAGAGGATGCTGGCTTCCGCTGGTGAGCTCCAGAAGGGTAACG
AGCTTGCTCTTCCTTCCAAGTACGTGAACTTCCTCTACCTCGCCTCCCACTACGAGAAG
CTCAAGGGTTCCCCTGAGGATAACGAGCAGAAGCAGCTCTTCGTGGAGCAGCACAAG
CACTACCTCGACGAGATCATCGAGCAGATCTCCGAGTTCTCCAAGCGCGTCATCCTCGC
TGACGCTAACCTCGACAAGGTCCTCTCCGCCTACAACAAGCACCGCGACAAGCCCATC
CGCGAGCAGGCCGAGAACATCATCCACCTCTTCACGCTCACGAACCTCGGCGCCCCTG
CTGCTTTCAAGTACTTCGACACCACCATCGACAGGAAGCGTTACACGTCCACCAAGGA
GGTTCTCGACGCTACTCTCATCCACCAGTCCATCACCGGTCTTTACGAGACTCGTATCG

ACCTTTCCCAGCTTGGTGGTGATAAGAGGCCTGCTGCTACTAAGAAGGCTGGACAAGC
TAAGAAGAAGAAGACTAGTTCAGGAGGATCTGGAGGTTCGGGTGGGTCCACGAACTT
GTCGGACATAATCGAGAAGGAAACAGGTAAACAACTCGTTATCCAAGAAAGCATTCTT
ATGTTGCCCGAGGAGGTTGAGGAAGTCATAGGAAACAAACCAGAGTCAGATATTCTCG
TTCATACCGCCTATGACGAATCAACAGATGAAAATGTGATGCTACTGACTTCTGATGCT
CCTGAGTACAAGCCATGGGCATTGGTGATACAGGACTCCAATGGAGAGAACAAAATAA
AAATGTTATCTGGTGGAAGTGGTGGCTCTGGCGGTTCAACGAATCTTAGCGATATCATT
GAGAAAGAAACTGGAAAACAGCTTGTGATTCAGGAGAGTATCCTGATGCTTCCTGAAG
AAGTTGAAGAGGTAATTGGGAACAAGCCTGAAAGTGACATTTTGGTTCACACTGCATA
TGATGAATCTACTGATGAGAATGTTATGTTACTAACAAGTGATGCGCCGGAATACAAAC
CTTGGGCTCTTGTCATTCAAGATTCTAATGGTGAAAACAAGATCAAGATGCTCAGCGG
GGGCTCCAAGAGAACCGCTGATGGATCAGAGTTTGAACCAAAGAAGAAAAGGAAAG
TACTAGTCCCTAGAGTCCTGCTTTAATGAGATATGCGAGACGCCTATGATCGCATGATAT
TTGCTTTCAATTCTGTTGTGCACGTTGTAAAAAACCTGAGCATGTGTAGCTCAGATCCT
TACCGCCGGTTTCGGTTCATTCTAATGAATATATCACCCGTTACTATCGTATTTTTATGAA
TAATATTCTCCGTTCAATTTACTGATTGTACCCTACTACTTATATGTACAATATTAAAATGA
AAACAATATATTGTGCTGAATAGGTTTATAGCGACATCTATGATAGAGCGCCACAATAAC
AAACAATTGCGTTTTATTATTACAAATCCAATTTTAAAAAAAGCGGCAGAACCGGTCAA
ACCTAAAAGACTGATTACATAAATCTTATTCAAATTTCAAAAGTGCCCCAGGGGCTAGT
ATCTACGACACACCGAGCGGCGAACTAATAACGCTCACTGAAGGGAACTCCGGTTCCC
CGCCGGCGCGCATGGGTGAGATTCCTTGAAGTTGAGTATTGGCCGTCCGCTCTACCGA
AAGTTACGGGCACCATTCAACCCGGTCCAGCACGGCGGCCGGGTAACCGACTTGCTGC
CCCGAGAATTATGCAGCATTTTTTTGGTGTATGTGGGCCCCAAATGAAGTGCAGGTCAA
ACCTTGACAGTGACGACAAATCGTTGGGCGGGTCCAGGGCGAATTTTGCGACAACATG
TCGAGGCTCAGCAGGAATTCGTCGTGCTCCACATGTTGACCGGTAAGGCGCGCCAAGC
TTCGTTGAACAACGGAAACTCGACTTGCCTTCCGCACAATACATCATTTCTTCTTAGCT
TTTTTTCTTCTTCTTCGTTCATACAGTTTTTTTTTGTTTATCAGCTTACATTTTCTTGAACC
GTAGCTTTCGTTTTCTTCTTTTTAACTTTCCATTCGGAGTTTTTGTATCTTGTTTCATAGT
TTGTCCCAGGATTAGAATGATTAGGCATCGAACCTTCAAGAATTTGATTGAATAAAACA
TCTTCATTCTTAAGATATGAAGATAATCTTCAAAAGGCCCCTGGGAATCTGAAAGAAGA
GAAGCAGGCCCATTTATATGGGAAAGAACAATAGTATTTCTTATATAGGCCCATTTAAGT
TGAAAACAATCTTCAAAAGTCCCACATCGCTTAGATAAGAAAACGAAGCTGAGTTTAT
ATACAGCTAGAGTCGAAGTAGTGATTGTCCCTTCGGAACAAAGCACCAGTGGTCTAGT
GGTAGAATAGTACCCTGCCACGGTACAGACCCGGGTTCGATTCCCGGCTGGTGCAAGA
GACCGGTCTCGGTTTCAGAGCTATGCTGGAAACAGCATAGCAAGTTGAAATAAGGCTA
GTCCGTTATCAACTTGAAAAAGTGGCACCGAGTCGGTGCTTTTTT
