# Supplementary material for: Cytosine base editors (CBEs) for inducing targeted DNA base editing in Nicotiana benthamiana
Source: BMC Plant Biol. 2023 Jun 7;23:305. doi: 10.1186/s12870-023-04322-8 (PMC10245509; doi:10.1186/s12870-023-04322-8)
Supplement: Supplementary file 10 — Additional file 10: Figure S10. The sequence of the rAPOBEC1(R33A)-CBE editing vector. Different colors represented different elements. [file 12870_2023_4322_MOESM10_ESM.pdf]

>rAPOBEC1(R33A)-CBE (35S promoter - 35aa linker – rAPOBEC1(R33A) - 20aa XTEN - nCas9- NLS - 2×UGI - NLS - OCS terminator - AtU6-26 - tRNA - BsaI - gRNA - Terminator)

Tgagacttttcaacaaagggtaatttcgggaaacctcctcggattccattgcccagctatctgtcacttcatcgaaaggacagtagaaaaggaa
ggtggctcctacaaatgccatcattgcgataaaggaaaggctatcattcaagatgcctctgccgacagtggtcccaaagatggacccccaccc
acgaggagcatcgtggaaaaagaagacgttccaaccacgtcttcaaagcaagtggattgatgtgacatctccactgacgtaagggatgacgc
acaatcccactatccttcgcaagacccttcctctatataaggaagttcatttcatttggagaggacagcccaagctgagctccaccgcggtggc
ggccgctctagaactagacaattaccaacaacaacaaacaacaaacaacattacaattacatttacaattacggatccATGTCCTCTG
AAACTGGACCTGTGGCCGTTGATCCTACTCTCCGCCGTAGAATTGAACCACATGAGTTT
GAAGTGTTTTTCGACCCGAGAGAACTCGCTAAAGAGACTTGCCTTCTGTATGAAATAA
ATTGGGGAGGAAGACACTCCATATGGAGACACACATCACAGAACACAAACAAACATG
TTGAGGTTAATTTCATCGAGAAGTTTACGACAGAGCGATACTTCTGCCCAAACACTCGG
TGTTCAATAACATGGTTCCTCAGCTGGTCCCCATGCGGAGAATGTAGCCGAGCAATCAC
AGAATTTTTATCAAGATATCCTCATGTCACGTTGTTTATTTATATCGCGAGACTCTATCAT
CATGCTGATCCACGGAACCGACAAGGTTTAAGGGATCTTATTTCGTCTGGAGTCACCAT
TCAGATCATGACTGAGCAGGAATCAGGTTATTGTTGGAGGAACTTTGTAAATTACTCTC
CGTCGAATGAAGCTCATTGGCCTCGTTACCCTCACCTTTGGGTTAGGCTTTATGTGTTA
GAGCTTTACTGTATTATACTAGGTCTGCCGCCTTGCTTGAATATTTTACGTAGGAAGCAA
CCACAATTGACTTTCTTCACAATCGCTCTTCAGTCTTGTCATTACCAAAGACTACCCCC
ACACATTTTGTGGGCAACTGGATTGAAGTCTGGATCTGAAACTCCTGGAACTTCTGAA
TCTGCTACTCCTGAATCTCTGCAGGGATCCGACAAGAAGTACTCCATCGGCCTCGCCAT
CGGCACCAACAGCGTCGGCTGGGCGGTGATCACCGACGAGTACAAGGTCCCGTCCAA
GAAGTTCAAGGTCCTGGGCAACACCGACCGCCACTCCATCAAGAAGAACCTCATCGG
CGCCCTCCTCTTCGACTCCGGCGAGACGGCGGAGGCGACCCGCCTCAAGCGCACCGC
CCGCCGCCGCTACACCCGCCGCAAGAACCGCATCTGCTACCTCCAGGAGATCTTCTCC
AACGAGATGGCGAAGGTCGACGACTCCTTCTTCCACCGCCTCGAGGAGTCCTTCCTCG
TGGAGGAGGACAAGAAGCACGAGCGCCACCCCATCTTCGGCAACATCGTCGACGAGG
TCGCCTACCACGAGAAGTACCCCACTATCTACCACCTTCGTAAGAAGCTTGTTGACTCT
ACTGATAAGGCTGATCTTCGTCTCATCTACCTTGCTCTCGCTCACATGATCAAGTTCCGT
GGTCACTTCCTTATCGAGGGTGACCTTAACCCTGATAACTCCGACGTGGACAAGCTCTT
CATCCAGCTCGTCCAGACCTACAACCAGCTCTTCGAGGAGAACCCTATCAACGCTTCC
GGTGTCGACGCTAAGGCGATCCTTTCCGCTAGGCTCTCCAAGTCCAGGCGTCTCGAGA
ACCTCATCGCCCAGCTCCCTGGTGAGAAGAAGAACGGTCTTTTCGGTAACCTCATCGC
TCTCTCCCTCGGTCTGACCCCTAACTTCAAGTCCAACTTCGACCTCGCTGAGGACGCTA
AGCTTCAGCTCTCCAAGGATACCTACGACGATGATCTCGACAACCTCCTCGCTCAGATT
GGAGATCAGTACGCTGATCTCTTCCTTGCTGCTAAGAACCTCTCCGATGCTATCCTCCTT
TCGGATATCCTTAGGGTTAACACTGAGATCACTAAGGCTCCTCTTTCTGCTTCCATGATC
AAGCGCTACGACGAGCACCACCAGGACCTCACCCTCCTCAAGGCTCTTGTTCGTCAGC
AGCTCCCCGAGAAGTACAAGGAGATCTTCTTCGACCAGTCCAAGAACGGCTACGCCG
GTTACATTGACGGTGGAGCTAGCCAGGAGGAGTTCTACAAGTTCATCAAGCCAATCCT
TGAGAAGATGGATGGTACTGAGGAGCTTCTCGTTAAGCTTAACCGTGAGGACCTCCTT
AGGAAGCAGAGGACTTTCGATAACGGCTCTATCCCTCACCAGATCCACCTTGGTGAGC
TTCACGCCATCCTTCGTAGGCAGGAGGACTTCTACCCTTTCCTCAAGGACAACCGTGA
GAAGATCGAGAAGATCCTTACTTTCCGTATTCCTTACTACGTTGGTCCTCTTGCTCGTGG
TAACTCCCGTTTCGCTTGGATGACTAGGAAGTCCGAGGAGACTATCACCCCTTGGAAC

TTCGAGGAGGTTGTTGACAAGGGTGCTTCCGCCCAGTCCTTCATCGAGCGCATGACCA
ACTTCGACAAGAACCTCCCCAACGAGAAGGTCCTCCCCAAGCACTCCCTCCTCTACGA
GTACTTCACGGTCTACAACGAGCTCACCAAGGTCAAGTACGTCACCGAGGGTATGCGC
AAGCCTGCCTTCCTCTCCGGCGAGCAGAAGAAGGCTATCGTTGACCTCCTCTTCAAGA
CCAACCGCAAGGTCACCGTCAAGCAGCTCAAGGAGGACTACTTCAAGAAGATCGAGT
GCTTCGACTCCGTCGAGATCAGCGGCGTTGAGGACCGTTTCAACGCTTCTCTCGGTAC
CTACCACGATCTCCTCAAGATCATCAAGGACAAGGACTTCCTCGACAACGAGGAGAAC
GAGGACATCCTCGAGGACATCGTCCTCACTCTTACTCTCTTCGAGGATAGGGAGATGAT
CGAGGAGAGGCTCAAGACTTACGCTCATCTCTTCGATGACAAGGTTATGAAGCAGCTC
AAGCGTCGCCGTTACACCGGTTGGGGTAGGCTCTCCCGCAAGCTCATCAACGGTATCA
GGGATAAGCAGAGCGGCAAGACTATCCTCGACTTCCTCAAGTCTGATGGTTTCGCTAA
CAGGAACTTCATGCAGCTCATCCACGATGACTCTCTTACCTTCAAGGAGGATATTCAGA
AGGCTCAGGTGTCCGGTCAGGGCGACTCTCTCCACGAGCACATTGCTAACCTTGCTGG
TTCCCCTGCTATCAAGAAGGGCATCCTTCAGACTGTTAAGGTTGTCGATGAGCTTGTCA
AGGTTATGGGTCGTCACAAGCCTGAGAACATCGTCATCGAGATGGCTCGTGAGAACCA
GACTACCCAGAAGGGTCAGAAGAACTCGAGGGAGCGCATGAAGAGGATTGAGGAGG
GTATCAAGGAGCTTGGTTCTCAGATCCTTAAGGAGCACCCTGTCGAGAACACCCAGCT
CCAGAACGAGAAGCTCTACCTCTACTACCTCCAGAACGGTAGGGATATGTACGTTGAC
CAGGAGCTCGACATCAACAGGCTTTCTGACTACGACGTCGACCACATTGTTCCTCAGT
CTTTCCTTAAGGATGACTCCATCGACAACAAGGTCCTCACGAGGTCCGACAAGAACAG
GGGTAAGTCGGACAACGTCCCTTCCGAGGAGGTTGTCAAGAAGATGAAGAACTACTG
GAGGCAGCTTCTCAACGCTAAGCTCATTACCCAGAGGAAGTTCGACAACCTCACGAA
GGCTGAGAGGGGTGGCCTTTCCGAGCTTGACAAGGCTGGTTTCATCAAGAGGCAGCT
TGTTGAGACGAGGCAGATTACCAAGCACGTTGCTCAGATCCTCGATTCTAGGATGAAC
ACCAAGTACGACGAGAACGACAAGCTCATCCGCGAGGTCAAGGTGATCACCCTCAAG
TCCAAGCTCGTCTCCGACTTCCGCAAGGACTTCCAGTTCTACAAGGTCCGCGAGATCA
ACAACTACCACCACGCTCACGATGCTTACCTTAACGCTGTCGTTGGTACCGCTCTTATC
AAGAAGTACCCTAAGCTTGAGTCCGAGTTCGTCTACGGTGACTACAAGGTCTACGACG
TTCGTAAGATGATCGCCAAGTCCGAGCAGGAGATCGGCAAGGCCACCGCCAAGTACTT
CTTCTACTCCAACATCATGAACTTCTTCAAGACCGAGATCACCCTCGCCAACGGCGAG
ATCCGCAAGCGCCCTCTTATCGAGACGAACGGTGAGACTGGTGAGATCGTTTGGGACA
AGGGTCGCGACTTCGCTACTGTTCGCAAGGTCCTTTCTATGCCTCAGGTTAACATCGTC
AAGAAGACCGAGGTCCAGACCGGTGGCTTCTCCAAGGAGTCTATCCTTCCAAAGAGA
AACTCGGACAAGCTCATCGCTAGGAAGAAGGATTGGGACCCTAAGAAGTACGGTGGT
TTCGACTCCCCTACTGTCGCCTACTCCGTCCTCGTGGTCGCCAAGGTGGAGAAGGGTA
AGTCGAAGAAGCTCAAGTCCGTCAAGGAGCTCCTCGGCATCACCATCATGGAGCGCTC
CTCCTTCGAGAAGAACCCGATCGACTTCCTCGAGGCCAAGGGCTACAAGGAGGTCAA
GAAGGACCTCATCATCAAGCTCCCCAAGTACTCTCTTTTCGAGCTCGAGAACGGTCGT
AAGAGGATGCTGGCTTCCGCTGGTGAGCTCCAGAAGGGTAACGAGCTTGCTCTTCCTT
CCAAGTACGTGAACTTCCTCTACCTCGCCTCCCACTACGAGAAGCTCAAGGGTTCCCC
TGAGGATAACGAGCAGAAGCAGCTCTTCGTGGAGCAGCACAAGCACTACCTCGACGA
GATCATCGAGCAGATCTCCGAGTTCTCCAAGCGCGTCATCCTCGCTGACGCTAACCTCG
ACAAGGTCCTCTCCGCCTACAACAAGCACCGCGACAAGCCCATCCGCGAGCAGGCCG
AGAACATCATCCACCTCTTCACGCTCACGAACCTCGGCGCCCCTGCTGCTTTCAAGTAC

TTCGACACCACCATCGACAGGAAGCGTTACACGTCCACCAAGGAGGTTCTCGACGCTA
CTCTCATCCACCAGTCCATCACCGGTCTTTACGAGACTCGTATCGACCTTTCCCAGCTT
GGTGGTGATAAGAGGCCTGCTGCTACTAAGAAGGCTGGACAAGCTAAGAAGAAGAAG
ACTAGTTCAGGAGGATCTGGAGGTTCGGGTGGGTCCACGAACTTGTCGGACATAATCG
AGAAGGAAACAGGTAAACAACTCGTTATCCAAGAAAGCATTCTTATGTTGCCCGAGGA
GGTTGAGGAAGTCATAGGAAACAAACCAGAGTCAGATATTCTCGTTCATACCGCCTATG
ACGAATCAACAGATGAAAATGTGATGCTACTGACTTCTGATGCTCCTGAGTACAAGCC
ATGGGCATTGGTGATACAGGACTCCAATGGAGAGAACAAAATAAAAATGTTATCTGGT
GGAAGTGGTGGCTCTGGCGGTTCAACGAATCTTAGCGATATCATTGAGAAAGAAACTG
GAAAACAGCTTGTGATTCAGGAGAGTATCCTGATGCTTCCTGAAGAAGTTGAAGAGGT
AATTGGGAACAAGCCTGAAAGTGACATTTTGGTTCACACTGCATATGATGAATCTACTG
ATGAGAATGTTATGTTACTAACAAGTGATGCGCCGGAATACAAACCTTGGGCTCTTGTC
ATTCAAGATTCTAATGGTGAAAACAAGATCAAGATGCTCAGCGGGGGCTCCAAGAGAA
CCGCTGATGGATCAGAGTTTGAACCAAAGAAGAAAAGGAAAGTACTAGTCCCTAGAG
TCCTGCTTTAATGAGATATGCGAGACGCCTATGATCGCATGATATTTGCTTTCAATTCTGT
TGTGCACGTTGTAAAAAACCTGAGCATGTGTAGCTCAGATCCTTACCGCCGGTTTCGGT
TCATTCTAATGAATATATCACCCGTTACTATCGTATTTTTATGAATAATATTCTCCGTTCAA
TTTACTGATTGTACCCTACTACTTATATGTACAATATTAAAATGAAAACAATATATTGTGC
TGAATAGGTTTATAGCGACATCTATGATAGAGCGCCACAATAACAAACAATTGCGTTTTA
TTATTACAAATCCAATTTTAAAAAAAGCGGCAGAACCGGTCAAACCTAAAAGACTGAT
TACATAAATCTTATTCAAATTTCAAAAGTGCCCCAGGGGCTAGTATCTACGACACACCG
AGCGGCGAACTAATAACGCTCACTGAAGGGAACTCCGGTTCCCCGCCGGCGCGCATG
GGTGAGATTCCTTGAAGTTGAGTATTGGCCGTCCGCTCTACCGAAAGTTACGGGCACC
ATTCAACCCGGTCCAGCACGGCGGCCGGGTAACCGACTTGCTGCCCCGAGAATTATGC
AGCATTTTTTTGGTGTATGTGGGCCCCAAATGAAGTGCAGGTCAAACCTTGACAGTGA
CGACAAATCGTTGGGCGGGTCCAGGGCGAATTTTGCGACAACATGTCGAGGCTCAGC
AGGAATTCGTCGTGCTCCACATGTTGACCGGTAAGGCGCGCCAAGCTTCGTTGAACAA
CGGAAACTCGACTTGCCTTCCGCACAATACATCATTTCTTCTTAGCTTTTTTTCTTCTTC
TTCGTTCATACAGTTTTTTTTTGTTTATCAGCTTACATTTTCTTGAACCGTAGCTTTCGTT
TTCTTCTTTTTAACTTTCCATTCGGAGTTTTTGTATCTTGTTTCATAGTTTGTCCCAGGAT
TAGAATGATTAGGCATCGAACCTTCAAGAATTTGATTGAATAAAACATCTTCATTCTTAA
GATATGAAGATAATCTTCAAAAGGCCCCTGGGAATCTGAAAGAAGAGAAGCAGGCCC
ATTTATATGGGAAAGAACAATAGTATTTCTTATATAGGCCCATTTAAGTTGAAAACAATC
TTCAAAAGTCCCACATCGCTTAGATAAGAAAACGAAGCTGAGTTTATATACAGCTAGAG
TCGAAGTAGTGATTGTCCCTTCGGAACAAAGCACCAGTGGTCTAGTGGTAGAATAGTA
CCCTGCCACGGTACAGACCCGGGTTCGATTCCCGGCTGGTGCAAGAGACCGGTCTCGG
TTTCAGAGCTATGCTGGAAACAGCATAGCAAGTTGAAATAAGGCTAGTCCGTTATCAAC
TTGAAAAAGTGGCACCGAGTCGGTGCTTTTTT
