# Supplementary material for: Cytosine base editors (CBEs) for inducing targeted DNA base editing in Nicotiana benthamiana
Source: BMC Plant Biol. 2023 Jun 7;23:305. doi: 10.1186/s12870-023-04322-8 (PMC10245509; doi:10.1186/s12870-023-04322-8)
Supplement: Supplementary file 11 — Additional file 11: Figure S11. The sequence of the slEF1α-A3A-CBE editing vector. Different colors represented different elements. [file 12870_2023_4322_MOESM11_ESM.pdf]

>*slEF1α*-A3A-CBE (*slEF1α* promoter - linker 1 - A3A - linker 2 - nCas9- NLS - 2×UGI - NLS - OCS terminator - AtU6-26 - tRNA - BsaI - gRNA - Terminator)

aagctagtcgacttttttttcttactgaaaattaatattttttcttttgaaatactaatacatctaaatttaacaattgccaaagtgatttttaattagcttg
ctggctaatcacaataaaaattactctcctttactatataagtaaatttttattgctatatttgttattattattattattaatatttattttctacaaatttaa
taatattttattttatatcattttaaaaagataagtaatgaaatattaagaattcgtttataattcttttgcaggtgggtttctatttgtaagctaatcttttttca
gttatcctttttttaaaatctttattattattatagctatatcttttatcttttaaaattaacattatctattaaagataatttcaataaaagagtaaaaattaatt
tagagttctactgtcttcaaatttctattttaaaaaatacttttaaaacttgatgtattttttacgtggtttttcactatgacttaatttctgttttattataatat
gtataaatataaaaatagattttccataacatattataaaaaatgtaaggggcatttacgtaaatagatagacttaaaagaggcaccgagtgaacc
ctaattctcatcgttgagactataaaatgcccattatcccattcgcacagtctcttcattacttttgctgttatttctcctcagctgtgccgcatatcgcc
taatttttcttctctaaggtttcatcatcttcaccaatttctttaatctcgattcaatttttatgtttgatctgttattgttctgtcactacatgtgtttttcagtt
gttttactagatgattttcactgtcttcttgttagatcatacatatattgaaaatgttttggattgactttttgtattgtgaatatctgttattgtttgattgttg
ttcagtatttacacacccgatctgtgttatgagcttggtcataactatttctctgtatgtaaatacagatctgttaatgtttgtaatcaattttcatatgca
ctgttgatattgttctctctcctgtcctgttatatgttgatatgattcggtttttgtataacttgaactaaacactagtcctaaatgtttttttactatttaag
atttatataatatggatagatttttgagttcctagtctctgaagaggttaagcttgctgtagttgtttaccagttgaggtgcaatactaaaaatcaattc
aattactgatatttttgctgtttaggtttttgacaaagtactttaatttgctttattgaactaaaaacgtagtcctgaattcattgcaagtgtgaaagctat
agttcattgtttttgttgcaattcttgaaaaattaattggtcaagctataatggattttactttttctgttttaatattgaatttgctgaatttatgaatgggtt
gcatggtttttgaaatatgttgttgtgtgttgtgtaaatgcagtttctttctagaactagacaattaccaacaacaacaaacaacaaacaacattaca
attacatttacaattacggatccATGGAAGCTTCTCCTGCTTCTGGACCTAGACATTTGATGGATCCTC
ATATTTTTACTTCTAATTTTAATAATGGAATTGGAAGACATAAGACTTATTTGTGTTATGA
AGTTGAAAGATTGGATAATGGAACTTCTGTTAAGATGGATCAACATAGAGGATTTTTGC
ATAATCAAGCTAAGAATTTGTTGTGTGGATTTTATGGAAGACATGCTGAATTGAGATTTT
TGGATTTGGTTCCTTCTTTGCAATTGGATCCTGCTCAAATTTATAGAGTTACTTGGTTTAT
TTCTTGGTCaCCTTGTTTTTCTTGGGGATGTGCTGGAGAAGTTAGAGCTTTTTTGCAAG
AAAATACTCATGTTAGATTGAGAATTTTTGCTGCTAGAATTTATGATTATGATCCTTTGTA
TAAGGAAGCTTTGCAAATGTTGAGAGATGCTGGAGCTCAAGTTTCTATTATGACTTATG
ATGAATTTAAGCATTGTTGGGATACTTTTGTTGATCATCAAGGATGTCCTTTTCAACCTT
GGGATGGATTGGATGAACATTCTCAAGCTTTGTCTGGAAGATTGAGAGCTATTTTGCAA
AATCAAGGAAATTCTGGATCTGAAACTCCTGGAACTTCTGAATCTGCTACTCCTGAATC
TCTGCAGGGATCCGACAAGAAGTACTCCATCGGCCTCGCCATCGGCACCAACAGCGTC
GGCTGGGCGGTGATCACCGACGAGTACAAGGTCCCGTCCAAGAAGTTCAAGGTCCTG
GGCAACACCGACCGCCACTCCATCAAGAAGAACCTCATCGGCGCCCTCCTCTTCGACT
CCGGCGAGACGGCGGAGGCGACCCGCCTCAAGCGCACCGCCCGCCGCCGCTACACCC
GCCGCAAGAACCGCATCTGCTACCTCCAGGAGATCTTCTCCAACGAGATGGCGAAGGT
CGACGACTCCTTCTTCCACCGCCTCGAGGAGTCCTTCCTCGTGGAGGAGGACAAGAA
GCACGAGCGCCACCCCATCTTCGGCAACATCGTCGACGAGGTCGCCTACCACGAGAA
GTACCCCACTATCTACCACCTTCGTAAGAAGCTTGTTGACTCTACTGATAAGGCTGATC
TTCGTCTCATCTACCTTGCTCTCGCTCACATGATCAAGTTCCGTGGTCACTTCCTTATCG
AGGGTGACCTTAACCCTGATAACTCCGACGTGGACAAGCTCTTCATCCAGCTCGTCCA
GACCTACAACCAGCTCTTCGAGGAGAACCCTATCAACGCTTCCGGTGTCGACGCTAAG
GCGATCCTTTCCGCTAGGCTCTCCAAGTCCAGGCGTCTCGAGAACCTCATCGCCCAGC
TCCCTGGTGAGAAGAAGAACGGTCTTTTCGGTAACCTCATCGCTCTCTCCCTCGGTCTG
ACCCCTAACTTCAAGTCCAACTTCGACCTCGCTGAGGACGCTAAGCTTCAGCTCTCCA
AGGATACCTACGACGATGATCTCGACAACCTCCTCGCTCAGATTGGAGATCAGTACGCT
GATCTCTTCCTTGCTGCTAAGAACCTCTCCGATGCTATCCTCCTTTCGGATATCCTTAGG

GTTAACACTGAGATCACTAAGGCTCCTCTTTCTGCTTCCATGATCAAGCGCTACGACGA
GCACCACCAGGACCTCACCCTCCTCAAGGCTCTTGTTCGTCAGCAGCTCCCCGAGAAG
TACAAGGAGATCTTCTTCGACCAGTCCAAGAACGGCTACGCCGGTTACATTGACGGTG
GAGCTAGCCAGGAGGAGTTCTACAAGTTCATCAAGCCAATCCTTGAGAAGATGGATGG
TACTGAGGAGCTTCTCGTTAAGCTTAACCGTGAGGACCTCCTTAGGAAGCAGAGGACT
TTCGATAACGGCTCTATCCCTCACCAGATCCACCTTGGTGAGCTTCACGCCATCCTTCG
TAGGCAGGAGGACTTCTACCCTTTCCTCAAGGACAACCGTGAGAAGATCGAGAAGATC
CTTACTTTCCGTATTCCTTACTACGTTGGTCCTCTTGCTCGTGGTAACTCCCGTTTCGCT
TGGATGACTAGGAAGTCCGAGGAGACTATCACCCCTTGGAACTTCGAGGAGGTTGTTG
ACAAGGGTGCTTCCGCCCAGTCCTTCATCGAGCGCATGACCAACTTCGACAAGAACCT
CCCCAACGAGAAGGTCCTCCCCAAGCACTCCCTCCTCTACGAGTACTTCACGGTCTAC
AACGAGCTCACCAAGGTCAAGTACGTCACCGAGGGTATGCGCAAGCCTGCCTTCCTCT
CCGGCGAGCAGAAGAAGGCTATCGTTGACCTCCTCTTCAAGACCAACCGCAAGGTCA
CCGTCAAGCAGCTCAAGGAGGACTACTTCAAGAAGATCGAGTGCTTCGACTCCGTCG
AGATCAGCGGCGTTGAGGACCGTTTCAACGCTTCTCTCGGTACCTACCACGATCTCCTC
AAGATCATCAAGGACAAGGACTTCCTCGACAACGAGGAGAACGAGGACATCCTCGAG
GACATCGTCCTCACTCTTACTCTCTTCGAGGATAGGGAGATGATCGAGGAGAGGCTCA
AGACTTACGCTCATCTCTTCGATGACAAGGTTATGAAGCAGCTCAAGCGTCGCCGTTAC
ACCGGTTGGGGTAGGCTCTCCCGCAAGCTCATCAACGGTATCAGGGATAAGCAGAGCG
GCAAGACTATCCTCGACTTCCTCAAGTCTGATGGTTTCGCTAACAGGAACTTCATGCAG
CTCATCCACGATGACTCTCTTACCTTCAAGGAGGATATTCAGAAGGCTCAGGTGTCCGG
TCAGGGCGACTCTCTCCACGAGCACATTGCTAACCTTGCTGGTTCCCCTGCTATCAAGA
AGGGCATCCTTCAGACTGTTAAGGTTGTCGATGAGCTTGTCAAGGTTATGGGTCGTCAC
AAGCCTGAGAACATCGTCATCGAGATGGCTCGTGAGAACCAGACTACCCAGAAGGGT
CAGAAGAACTCGAGGGAGCGCATGAAGAGGATTGAGGAGGGTATCAAGGAGCTTGGT
TCTCAGATCCTTAAGGAGCACCCTGTCGAGAACACCCAGCTCCAGAACGAGAAGCTCT
ACCTCTACTACCTCCAGAACGGTAGGGATATGTACGTTGACCAGGAGCTCGACATCAA
CAGGCTTTCTGACTACGACGTCGACCACATTGTTCCTCAGTCTTTCCTTAAGGATGACT
CCATCGACAACAAGGTCCTCACGAGGTCCGACAAGAACAGGGGTAAGTCGGACAACG
TCCCTTCCGAGGAGGTTGTCAAGAAGATGAAGAACTACTGGAGGCAGCTTCTCAACG
CTAAGCTCATTACCCAGAGGAAGTTCGACAACCTCACGAAGGCTGAGAGGGGTGGCC
TTTCCGAGCTTGACAAGGCTGGTTTCATCAAGAGGCAGCTTGTTGAGACGAGGCAGAT
TACCAAGCACGTTGCTCAGATCCTCGATTCTAGGATGAACACCAAGTACGACGAGAAC
GACAAGCTCATCCGCGAGGTCAAGGTGATCACCCTCAAGTCCAAGCTCGTCTCCGACT
TCCGCAAGGACTTCCAGTTCTACAAGGTCCGCGAGATCAACAACTACCACCACGCTCA
CGATGCTTACCTTAACGCTGTCGTTGGTACCGCTCTTATCAAGAAGTACCCTAAGCTTG
AGTCCGAGTTCGTCTACGGTGACTACAAGGTCTACGACGTTCGTAAGATGATCGCCAA
GTCCGAGCAGGAGATCGGCAAGGCCACCGCCAAGTACTTCTTCTACTCCAACATCATG
AACTTCTTCAAGACCGAGATCACCCTCGCCAACGGCGAGATCCGCAAGCGCCCTCTTA
TCGAGACGAACGGTGAGACTGGTGAGATCGTTTGGGACAAGGGTCGCGACTTCGCTA
CTGTTCGCAAGGTCCTTTCTATGCCTCAGGTTAACATCGTCAAGAAGACCGAGGTCCA
GACCGGTGGCTTCTCCAAGGAGTCTATCCTTCCAAAGAGAAACTCGGACAAGCTCATC
GCTAGGAAGAAGGATTGGGACCCTAAGAAGTACGGTGGTTTCGACTCCCCTACTGTCG
CCTACTCCGTCCTCGTGGTCGCCAAGGTGGAGAAGGGTAAGTCGAAGAAGCTCAAGT

CCGTCAAGGAGCTCCTCGGCATCACCATCATGGAGCGCTCCTCCTTCGAGAAGAACCC
GATCGACTTCCTCGAGGCCAAGGGCTACAAGGAGGTCAAGAAGGACCTCATCATCAA
GCTCCCCAAGTACTCTCTTTTCGAGCTCGAGAACGGTCGTAAGAGGATGCTGGCTTCC
GCTGGTGAGCTCCAGAAGGGTAACGAGCTTGCTCTTCCTTCCAAGTACGTGAACTTCC
TCTACCTCGCCTCCCACTACGAGAAGCTCAAGGGTTCCCCTGAGGATAACGAGCAGAA
GCAGCTCTTCGTGGAGCAGCACAAGCACTACCTCGACGAGATCATCGAGCAGATCTCC
GAGTTCTCCAAGCGCGTCATCCTCGCTGACGCTAACCTCGACAAGGTCCTCTCCGCCT
ACAACAAGCACCGCGACAAGCCCATCCGCGAGCAGGCCGAGAACATCATCCACCTCT
TCACGCTCACGAACCTCGGCGCCCCTGCTGCTTTCAAGTACTTCGACACCACCATCGA
CAGGAAGCGTTACACGTCCACCAAGGAGGTTCTCGACGCTACTCTCATCCACCAGTCC
ATCACCGGTCTTTACGAGACTCGTATCGACCTTTCCCAGCTTGGTGGTGATAAGAGGCC
TGCTGCTACTAAGAAGGCTGGACAAGCTAAGAAGAAGAAGACTAGTTCAGGAGGATC
TGGAGGTTCGGGTGGGTCCACGAACTTGTCGGACATAATCGAGAAGGAAACAGGTAA
ACAACTCGTTATCCAAGAAAGCATTCTTATGTTGCCCGAGGAGGTTGAGGAAGTCATA
GGAAACAAACCAGAGTCAGATATTCTCGTTCATACCGCCTATGACGAATCAACAGATG
AAAATGTGATGCTACTGACTTCTGATGCTCCTGAGTACAAGCCATGGGCATTGGTGATA
CAGGACTCCAATGGAGAGAACAAAATAAAAATGTTATCTGGTGGAAGTGGTGGCTCTG
GCGGTTCAACGAATCTTAGCGATATCATTGAGAAAGAAACTGGAAAACAGCTTGTGAT
TCAGGAGAGTATCCTGATGCTTCCTGAAGAAGTTGAAGAGGTAATTGGGAACAAGCCT
GAAAGTGACATTTTGGTTCACACTGCATATGATGAATCTACTGATGAGAATGTTATGTTA
CTAACAAGTGATGCGCCGGAATACAAACCTTGGGCTCTTGTCATTCAAGATTCTAATGG
TGAAAACAAGATCAAGATGCTCAGCGGGGGCTCCAAGAGAACCGCTGATGGATCAGA
GTTTGAACCAAAGAAGAAAAGGAAAGTACTAGTCCCTAGAGTCCTGCTTTAATGAGAT
ATGCGAGACGCCTATGATCGCATGATATTTGCTTTCAATTCTGTTGTGCACGTTGTAAAA
AACCTGAGCATGTGTAGCTCAGATCCTTACCGCCGGTTTCGGTTCATTCTAATGAATATA
TCACCCGTTACTATCGTATTTTTATGAATAATATTCTCCGTTCAATTTACTGATTGTACCCT
ACTACTTATATGTACAATATTAAAATGAAAACAATATATTGTGCTGAATAGGTTTATAGCG
ACATCTATGATAGAGCGCCACAATAACAAACAATTGCGTTTTATTATTACAAATCCAATT
TTAAAAAAAGCGGCAGAACCGGTCAAACCTAAAAGACTGATTACATAAATCTTATTCA
AATTTCAAAAGTGCCCCAGGGGCTAGTATCTACGACACACCGAGCGGCGAACTAATAA
CGCTCACTGAAGGGAACTCCGGTTCCCCGCCGGCGCGCATGGGTGAGATTCCTTGAAG
TTGAGTATTGGCCGTCCGCTCTACCGAAAGTTACGGGCACCATTCAACCCGGTCCAGC
ACGGCGGCCGGGTAACCGACTTGCTGCCCCGAGAATTATGCAGCATTTTTTTGGTGTAT
GTGGGCCCCAAATGAAGTGCAGGTCAAACCTTGACAGTGACGACAAATCGTTGGGCG
GGTCCAGGGCGAATTTTGCGACAACATGTCGAGGCTCAGCAGGAATTCGTCGTGCTCC
ACATGTTGACCGGTAAGGCGCGCCAAGCTTCGTTGAACAACGGAAACTCGACTTGCCT
TCCGCACAATACATCATTTCTTCTTAGCTTTTTTTCTTCTTCTTCGTTCATACAGTTTTTT
TTTGTTTATCAGCTTACATTTTCTTGAACCGTAGCTTTCGTTTTCTTCTTTTTAACTTTCC
ATTCGGAGTTTTTGTATCTTGTTTCATAGTTTGTCCCAGGATTAGAATGATTAGGCATCG
AACCTTCAAGAATTTGATTGAATAAAACATCTTCATTCTTAAGATATGAAGATAATCTTC
AAAAGGCCCCTGGGAATCTGAAAGAAGAGAAGCAGGCCCATTTATATGGGAAAGAAC
AATAGTATTTCTTATATAGGCCCATTTAAGTTGAAAACAATCTTCAAAAGTCCCACATCG
CTTAGATAAGAAAACGAAGCTGAGTTTATATACAGCTAGAGTCGAAGTAGTGATTGTCC
CTTCGGAACAAAGCACCAGTGGTCTAGTGGTAGAATAGTACCCTGCCACGGTACAGAC

CCGGGTTCGATTCCCGGCTGGTGCAAGAGACCGGTCTCGGTTTCAGAGCTATGCTGGA
AACAGCATAGCAAGTTGAAATAAGGCTAGTCCGTTATCAACTTGAAAAAGTGGCACCG
AGTCGGTGCTTTTTT
