# Supplementary material for: Cytosine base editors (CBEs) for inducing targeted DNA base editing in Nicotiana benthamiana
Source: BMC Plant Biol. 2023 Jun 7;23:305. doi: 10.1186/s12870-023-04322-8 (PMC10245509; doi:10.1186/s12870-023-04322-8)
Supplement: Supplementary file 3 — Additional file 3: Figure S3. The editing efficiencies of A3A-CBE, A3A (Y130F)-CBE, and rAPOBEC1(R33A)-CBE for converting C-to-A at (A) T1 ~ T7 target sites, and (B) T8 ~ T14 target sites. The editing efficiencies were calculated from three independent replicates' deep sequencing analysis results. [file 12870_2023_4322_MOESM3_ESM.pdf]

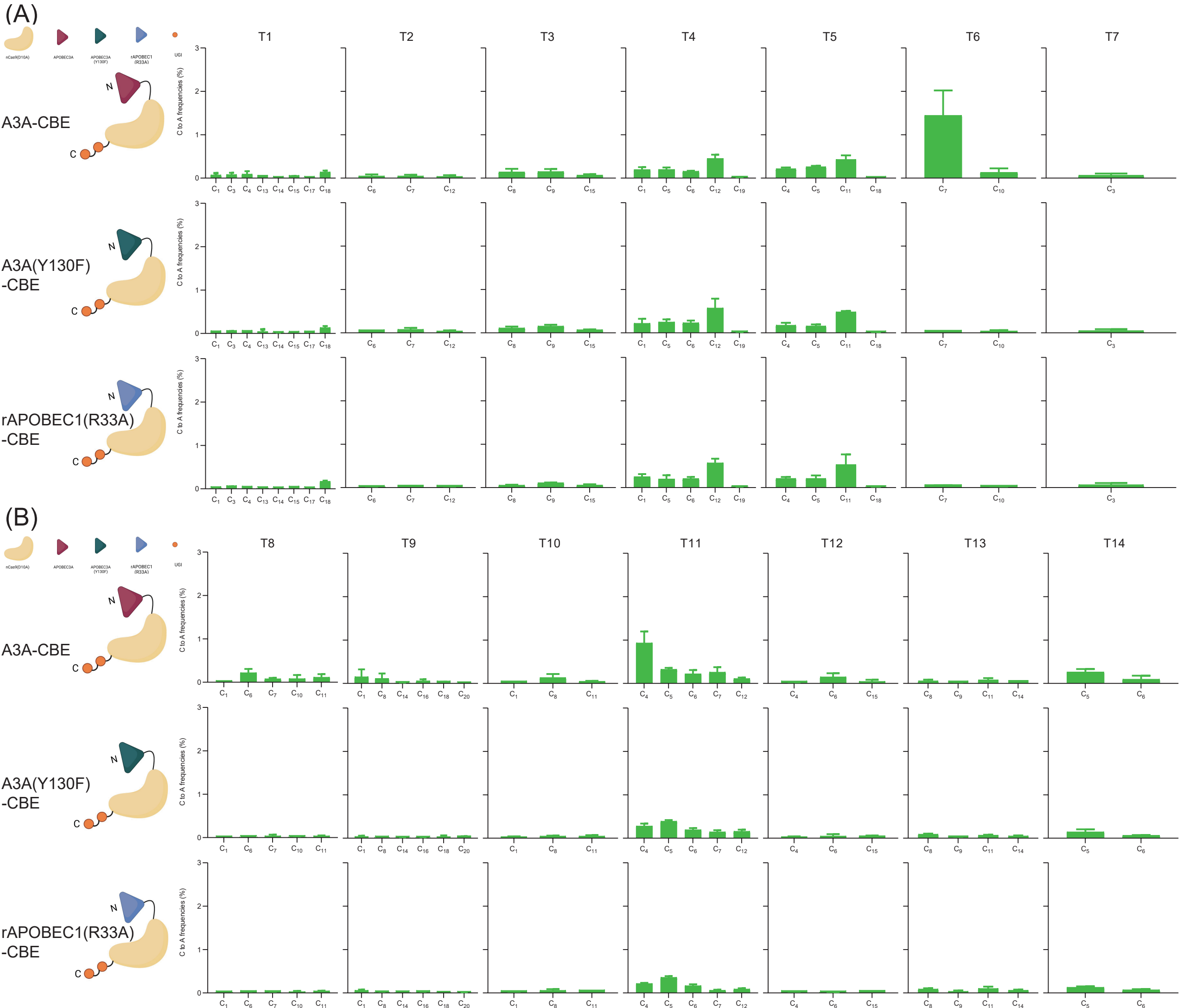

(A)
nCas9(D10A)
APOBEC3A
APOBEC3A (Y130F)
rAPOBEC1 (R33A)
UGI
A3A-CBE
A3A(Y130F) -CBE
rAPOBEC1(R33A) -CBE
N
C
C to A frequencies (%)
T1
T2
T3
T4
T5
T6
T7
C1 C3 C4 C13 C14 C15 C17 C18
C6 C7 C12
C8 C9 C15
C1 C5 C6 C12 C19
C4 C5 C11 C18
C7 C10
C3
(B)
T8
T9
T10
T11
T12
T13
T14
C1 C6 C7 C10 C11
C1 C8 C14 C16 C18 C20
C1 C8 C11
C4 C5 C6 C7 C12
C4 C6 C15
C8 C9 C11 C14
C5 C6
